# Targeting IL-3Rα on tumor-derived endothelial cells blunts metastatic spread of triple-negative breast cancer via extracellular vesicle reprogramming

**DOI:** 10.1038/s41389-020-00274-y

**Published:** 2020-10-10

**Authors:** Tatiana Lopatina, Cristina Grange, Claudia Cavallari, Victor Navarro-Tableros, Giusy Lombardo, Arturo Rosso, Massimo Cedrino, Margherita Alba Carlotta Pomatto, Malvina Koni, Francesca Veneziano, Isabella Castellano, Giovanni Camussi, Maria Felice Brizzi

**Affiliations:** 1 Department of Medical Sciences, University of Turin, Turin, Italy; 2 2i3T Scarl University of Turin, Turin, Italy

**Keywords:** Breast cancer, Drug development

## Abstract

The lack of approved targeted therapies highlights the need for new treatments for triple-negative breast cancer (TNBC) patients. Interleukin-3 (IL-3) acts as an autocrine factor for tumor–endothelial cells (TEC), and exerts pro-angiogenic paracrine action via extracellular vesicles (EVs). IL-3Rα blockade on TEC changes TEC-EV (anti-IL-3R-EV) microRNA (miR) content and promotes the regression of established vessels. As TEC is the doorway for “drug” entry into tumors, we aimed to assess whether IL-3R blockade on TEC impacts tumor progression via its unique EV cargo. First, the expression of IL-3Rα was evaluated in 27 human TNBC samples. It was noticed that, besides TEC and inflammatory cells, tumor cells from 55.5% of the human TNBC samples expressed IL-3Rα. Using human TNBC cell lines for in vitro studies, we found that, unlike native TEC-EVs (nEVs), anti-IL-3R-EVs increase apoptosis and reduced cell viability and migration. In vivo, anti-IL-3R-EV treatment induced vessel regression in established tumors formed of MDA-MB-231 cells, decreased Vimentin, β-catenin, and TWIST1 expression, almost abolished liver and lung metastases from primary tumors, and reduced lung metastasis generated via the intravenous injection of MDA-MB-231 cells. nEVs depleted of miR-24-3p (antago-miR-24-3p-EVs) were effective as anti-IL-3R-EVs in downregulating TWIST1 and reducing metastatic lesions in vivo. Consistent with network analyses of miR-24-3p gene targeting, anti-IL-3R-EVs and antago-miR-24-3p-EVs upregulate SPRY2 in MDA-MB-231 cells. Finally, SPRY2 silencing prevented anti-IL-3R-EV and antago-miR-24-3p-EV-mediated apoptotic cues.

Overall, these data provide the first evidence that IL-3Rα is highly expressed in TNBC cells, TEC, and inflammatory cells, and that IL-3Rα blockade on TEC impacts tumor progression.

## Introduction

Interleukin-3 (IL-3), a cytokine mainly produced by activated T and mast cells, is involved in the regulation of hemopoietic pluripotent and progenitor cell expansion^1^. Moreover, the role of IL-3 in controlling the proliferation/survival of various target cells, including normal and tumor-derived endothelial cells (TEC), has also been reported^2,3^. IL-3 binding to its receptor promotes numerous biological effects by regulating the expression of proteins, transcriptional factors^4,5^, and regulatory noncoding RNA, such as microRNAs (miRs)^6,7^.

IL-3 has seen most study in hematologic malignancies^8,9^. However, the observation that tumor-infiltrating lymphocytes (TILs)^10^ and TEC are able to produce IL-3^11^, sustains the possibility that IL-3 can also control the tumor microenvironment (TME). IL-3 acts as an autocrine pro-survival factor, particularly in TEC^11^. It is widely accepted that the autocrine mechanism of growth, increased AKT signaling pathway activation^12,13^, and the expression of pro-tumorigenic and angiogenic receptors and proteins reflects the unique TEC phenotype, which is distinct from that of normal endothelial cells^14–17^. Moreover, TEC, besides providing oxygen and nutrient supply, regulates tumor cell viability and the epithelial–mesenchymal transition (EMT) in the TME^18^. EMT is a highly regulated process that occurs during developmental processes and contributes to chemoresistance and metastasis^19^. A number of different transcriptional factors, including the zinc finger enhancer (E)-box-binding homeobox (ZEB), SNAIL, and TWIST1, strictly control EMT^20^. Moreover, there is considerable evidence for the interplay between these transcriptional factors and miRs coordinating the entire EMT process^20^. Cancer aggressiveness has also been associated with the ability of cancer cells to build their own vascular network without recruiting endothelial cells, a process denoted as vasculogenic mimicry (VM)^21,22^.

Triple-negative breast cancer (TNBC) is the most aggressive and prevalent subtype of breast cancer in women worldwide. Chemotherapy is still the main therapeutic approach at the early stage, as no approved targeted therapy for TNBC is currently available^23^. Tumor initiation, metastasis, relapse, and therapeutic resistance are triggered by dynamic changes in tumors that mainly depend on the conditions to which tumors are usually exposed, and on cell-to-cell communication in the TME, which occurs via soluble mediators and extracellular vesicles (EVs)^24^. EVs regulate cell-to-cell communication both locally in the TME and at distant sites^25^. EVs are complex multifunctional structures containing receptors, growth factors, other proteins, and different types of RNA^26^. It has been shown that EV molecular composition and functions depend on numerous cues, including those emanated inside TME by different cell types^27,28^. For example, tumor-derived EVs carrying pro-tumorigenic proteins, such as transcription factors, miRs, and growth factors, strictly control tumor growth and metastasis^29,30^. Moreover, EVs released by TECs (TEC-EVs) acquire unique miR-EV cargo, granting them their paracrine pro-angiogenic properties^31^.

Antibody-based anticancer therapy is currently seen as one of the most successful strategies for the treatment of both hematologic and solid tumors^32^. Monoclonal antibodies (mAbs) can directly act on tumor cells, induce cell killing by immune-mediated mechanisms, and specifically interfere with tumor vasculature and stromal cells. IL-3Rα is highly expressed in hematological malignant cells^33,34^, and its expression translates into blast proliferation, increased cellularity, and poor prognosis^35^. Therefore, the anti-IL-3Rα antibody has been proposed, and a Phase I clinical trial in patients with acute myeloid leukemia has demonstrated its safety^36^.

We have recently provided evidence that blocking IL-3Rα (anti-CD123mAb) on TEC leads to the release of EVs (anti-IL-3R-EVs) that display antiangiogenic properties^31^. In particular, we have shown that the IL-3Rα blockade changes EV miR composition, translating into the inhibition of the Wnt/β-catenin pathway. The loss of miR-24-3p was found to be crucial in mediating anti-IL-3R-EV vessel regression in vivo. Since TEC is the gateway to tumors, we sought to determine whether IL-3Rα blockade on TEC could challenge tumors and hamper progression via their reprogrammed EVs.

## Results

### Human TNBC expresses the IL-3Rα in TME

**Fig. 1:**
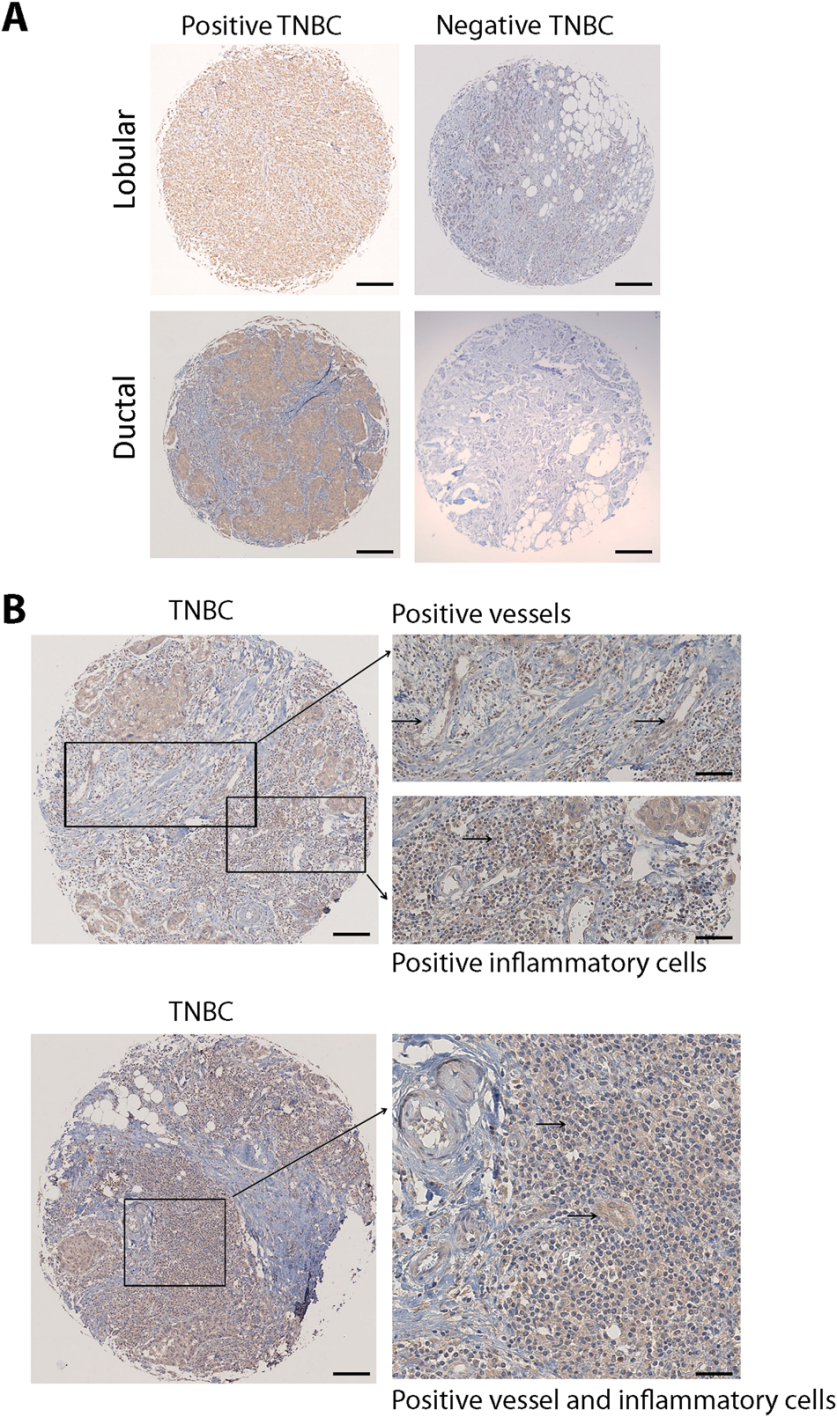
IL-3Rα immunoreactivity on human TNBC samples. Representative tissue microarrays obtained from human TNBC samples stained with anti-IL-3Rα antibody (*n* = 27). **a** Positive and negative expression of anti-IL-3Rα in neoplastic cells (ductal and lobular TNBC samples as indicated). Original magnification 40×, scale bar: 200 µm. **b** Focal and moderate IL-3Rα immunoreactivity of stromal inflammatory and endothelial cells indicated by arrows. Original magnification, 100× and 150×, scale bar: 100 µm and 75 µm, respectively.

Mesenchymal and mesenchymal stem-like subtypes of TNBC tumors have recently been associated with high angiogenetic signatures^37^. Since IL-3 is released in TME^10^ and acts as an autocrine growth factor for breast and renal TEC^11^, the expression of its binding subunit, IL-3Rα, was analyzed in 27 TNBC human samples. Supplementary Table S1 reports human TNBC features. As shown in Fig. 1, immunohistochemical analysis demonstrated that IL-3Rα is expressed by inflammatory cells and TEC. Interestingly, tumor cells also expressed IL-3Rα in 15 out of 27 (55.5%) samples. To confirm these data, IL-3Rα was also evaluated in the TNBC cell lines, MDA-MB-231 and MDA-MB-453, and in the nonneoplastic breast cancer cell line, MCF10A. As shown in Supplementary Fig. S1, TNBC cell lines, but not MCF10A, express IL-3Rα.

Since TEC in TNBC expresses IL-3Rα and TEC targeted by the anti-IL-3Rα antibody release paracrine signals that induce vessel regression^31^, we hypothesize that IL-3Rα blockade on TEC, via EVs, would be effective in driving dynamic changes in tumors/TME interfering with cancer progression.

### Anti-IL-3R-EVs, unlike naive EVs (nEVs), reduce cell number and migration and increase apoptosis of TNBC cell lines

**Fig. 2:**
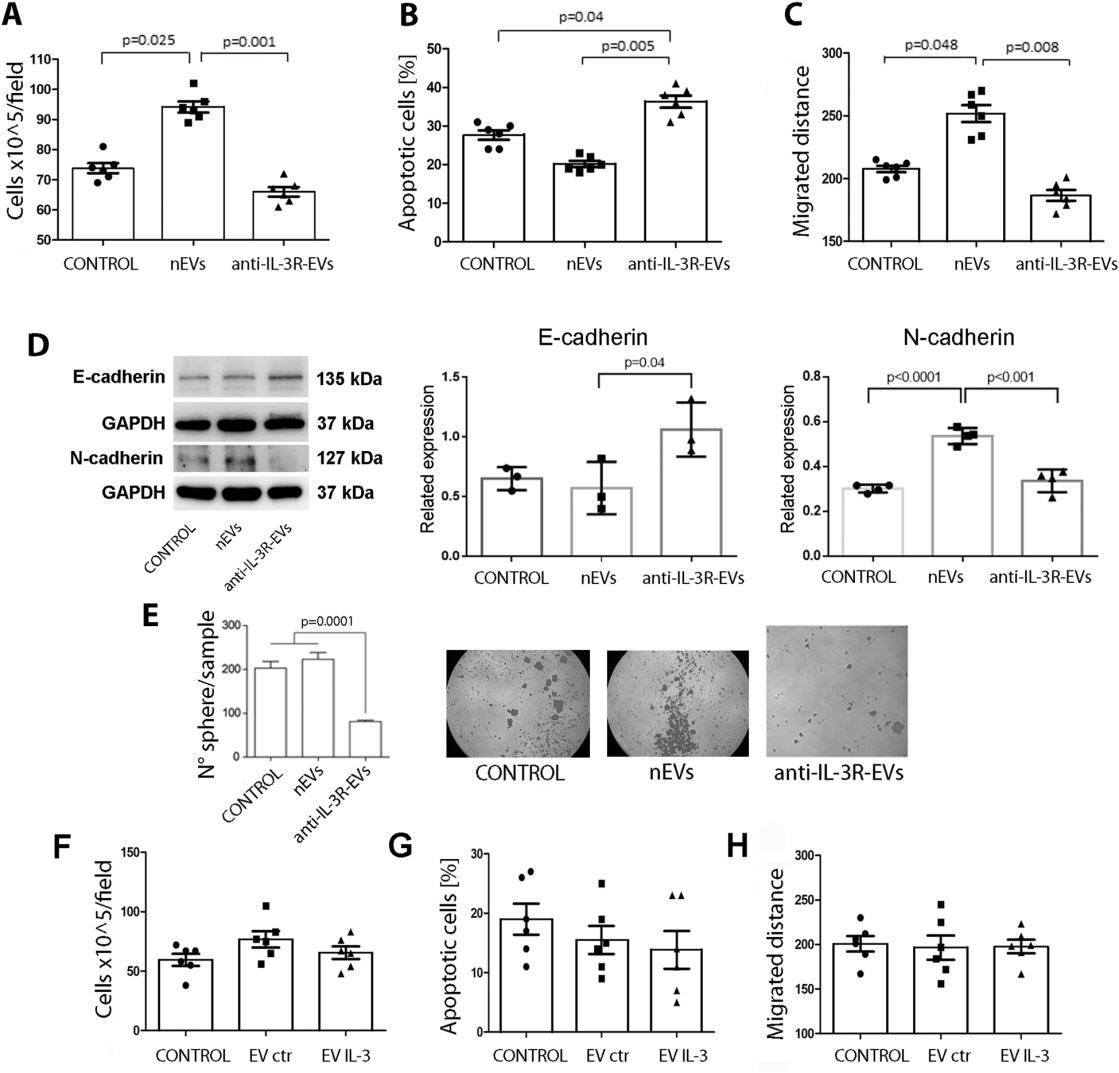
In vitro effects of anti-IL-3R-EVs on MDA-MB-231 cells. **a** Cells were stimulated for 48 h with nEVs or anti-IL-3R-EVs (data are expressed as mean ± SD) (*n* = 6). **b** To evaluate the apoptotic rate, cells were stimulated with nEVs and anti-IL-3R-EVs for 24 h (data are expressed as the percentage of apoptotic cells ± SD) (*n* = 6). **c** Migration assay. Data are expressed as distance ± SD of cells stimulated with nEVs and anti-IL-3R-EVs (*n* = 6). **d** Representative western blot and quantification of E- and N-cadherin expression in MDA-MB-231 cells untreated (CONTROL) or treated with TEC-EVs (nEVs or anti-IL-3R-EVs). Data are expressed as the mean ± SD normalized to GAPDH (*n* = 3). **e** Representative micrographs and histogram reporting data of the sphere formed by MDA-MB-231 cells stimulated with nEVs and anti-IL-3R-EVs (original magnification 40×). **f**–**h** Cell number (**f**), apoptosis (**g**), and migration (**h**) of MDA-MB-231 cells after stimulation with EVs derived from normal endothelial cells (HUVEC) untreated (EV ctr) or treated with IL-3 (EV IL-3) for 48 h (**f**), 24 h (**g**), and 24 h (**h**) (*n* = 6).

Naive EVs derived from TEC (nEVs) and anti-IL-3R-EVs were isolated from TEC and analyzed by TEM (Supplementary Fig. S2A) and NanoSight (data not shown). No differences in nEV and anti-IL-3R-EV size were detected. Fluorescence-activated cell sorting (FACS) analysis, using the MACSPlex exosome kit, revealed a similar pattern of surface marker expression. They expressed exosomal markers (CD9, CD63, and CD81) (Supplementary Fig. S2C) and integrins (CD49e/Integrin α-5 and CD29/Integrin β-1). The CD63 exosomal marker was also demonstrated by western blot (Supplementary Fig. S2B). Therefore, their effects were first evaluated on MDA-MB-231 and MDA-MB-453 cell lines in vitro. We demonstrated that, while nEVs were effective in increasing cell number, anti-IL-3R-EVs significantly reduced their number compared to untreated and nEV-treated cells (Fig. 2a, Supplementary Fig. S3A). Apoptosis and cell migration were also evaluated. Unlike nEV-, anti-IL-3R-EV treatment increased the number of apoptotic cells, and significantly reduced cell migration (Fig. 2b, c, Supplementary Fig. S3B, C). These results were also supported by the expression of E- and N-cadherin (Fig. 2d) and by the in vitro sphere-formation assay of nEVs and anti-IL-3R-EV-treated MDA-MB-231 cells (Fig. 2e). nEVs and anti-IL-3R-EVs were ineffective in inducing proliferation of MCF10A cells (Supplementary Fig. S3D). Overall, these results suggested that nEVs boost tumor cell growth/migration, while anti-IL-3R-EVs induce inhibition of cell growth and migration, and drive apoptosis. To evaluate whether this effect specifically relied on the abnormal TEC phenotype, EVs released by normal endothelial cells (EC) exposed to IL-3 (EV IL-3) were evaluated in tumor cells. Naive EC-derived EVs (EV ctr) served as controls. As shown in Fig. 2f–h, EV IL-3 failed to increase tumor cell number, its migration, or apoptotic rate. This indicates that the pro-tumorigenic action of nEVs mainly relies on TEC unique phenotype.

### Anti-IL-3R-EVs impair tumor angiogenesis and the formation of lung and liver metastasis of established tumors

**Fig. 3:**
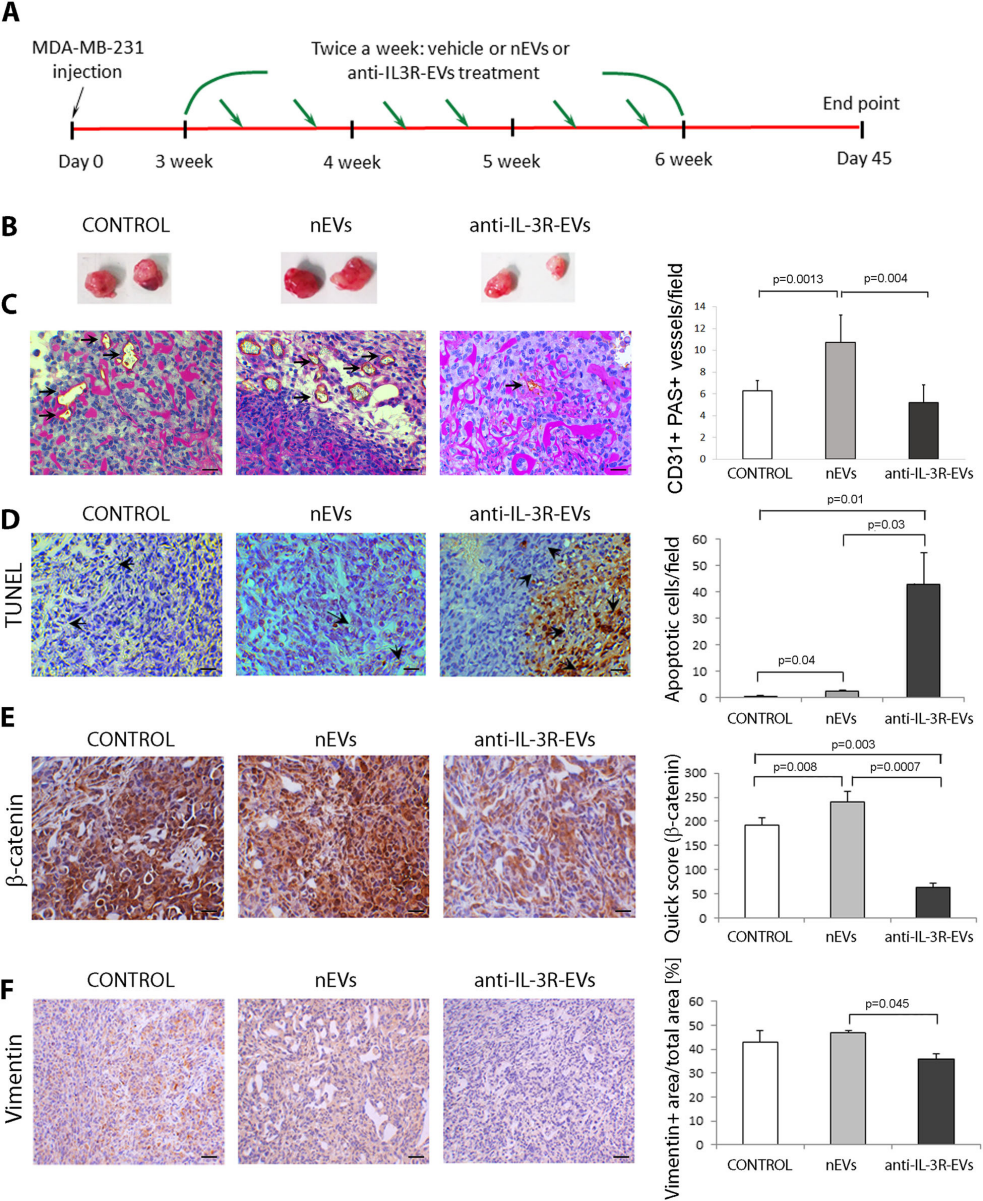
Effects of nEV and anti-IL-3R-EV treatment on MDA-MB-231-derived in vivo tumors. **a** Schematic representation of the experimental design to test TEC-EVs (nEVs and anti-IL-3R-EVs) on MDA-MB-231-derived tumors. **b** Representative images of tumors untreated (CONTROL) or treated with TEC-EVs (nEVs and anti-IL-3R-EVs) (*n* = 4). **c** Representative images of tumors untreated (CONTROL) or treated with TEC-EVs (nEVs and anti-IL-3R-EVs) stained with anti-CD31 antibody and PAS. Vessels within MDA-MB-231 tumors were expressed as the number of CD31+/PAS+ per field ± SD (*n* = 4). Original magnification 400×, scale bar: 25 µm. **d** Representative micrographs showing apoptosis within tumors stained using the Tunel assay. Quantification of tumor apoptosis expressed as the number of apoptotic cells/field (*n* = 4). Original magnification 400×, scale bar: 25 µm. **e** Representative immunohistochemical images of β-catenin-positive staining of MDA-MB-231-derived tumors of animals that had been left untreated (CONTROL) or treated with TEC-EVs (nEVs and anti-IL-3R-EVs). Quantification of β-catenin positivity was calculated using the Quick score ± SD (*n* = 4). Original magnification 400×, scale bar: 25 µm. **f** Representative immunohistochemical images of Vimentin-positive staining on MDA-MB-231-derived tumors from animals that had been left untreated (CONTROL) or treated with TEC-EVs (nEVs and anti-IL-3R-EVs). Quantification of Vimentin-positive area expressed as the percentage of Vimentin + area/total area ± SD (*n* = 4). Original magnification 200×, scale bar: 50 µm.

To investigate the effects of nEVs and anti-IL-3R-EVs in vivo, MDA-MB-231 cells were used for mammary fat pad injection into SCID mice. After 3 weeks when palpable tumors were detected, vehicle, nEVs, or anti-IL-3R-EVs were locally injected twice a week and the tumors followed for an additional 21 days (Fig. 3a). Mice were sacrificed at day 45, and primary tumors, the liver and lung, were analyzed by histology. As shown by the analysis of tumor vascular density, tumors from animals treated with anti-IL-3R-EVs displayed significantly reduced CD31-positive vessels (Fig. 3b, c, Supplementary Fig. S4). Moreover, a slight, but not significant reduction of PAS-positive/CD31-negative vessels, corresponding to the vascular network built by tumor cells (VM), was observed upon anti-IL-3R-EV treatment (Supplementary Fig. S5). Accordingly, increased apoptosis was found in the tumors of animals treated with anti-IL-3R-EVs (Fig. 3d). Of note, when compared to control animals, we found an increased apoptotic rate in tumors from animals treated with nEVs. Although we do not have direct pieces of evidence, we can speculate that hypoxia or depletion of survival factors may suppress apoptotic cues in control tumors^38^.

**Fig. 4:**
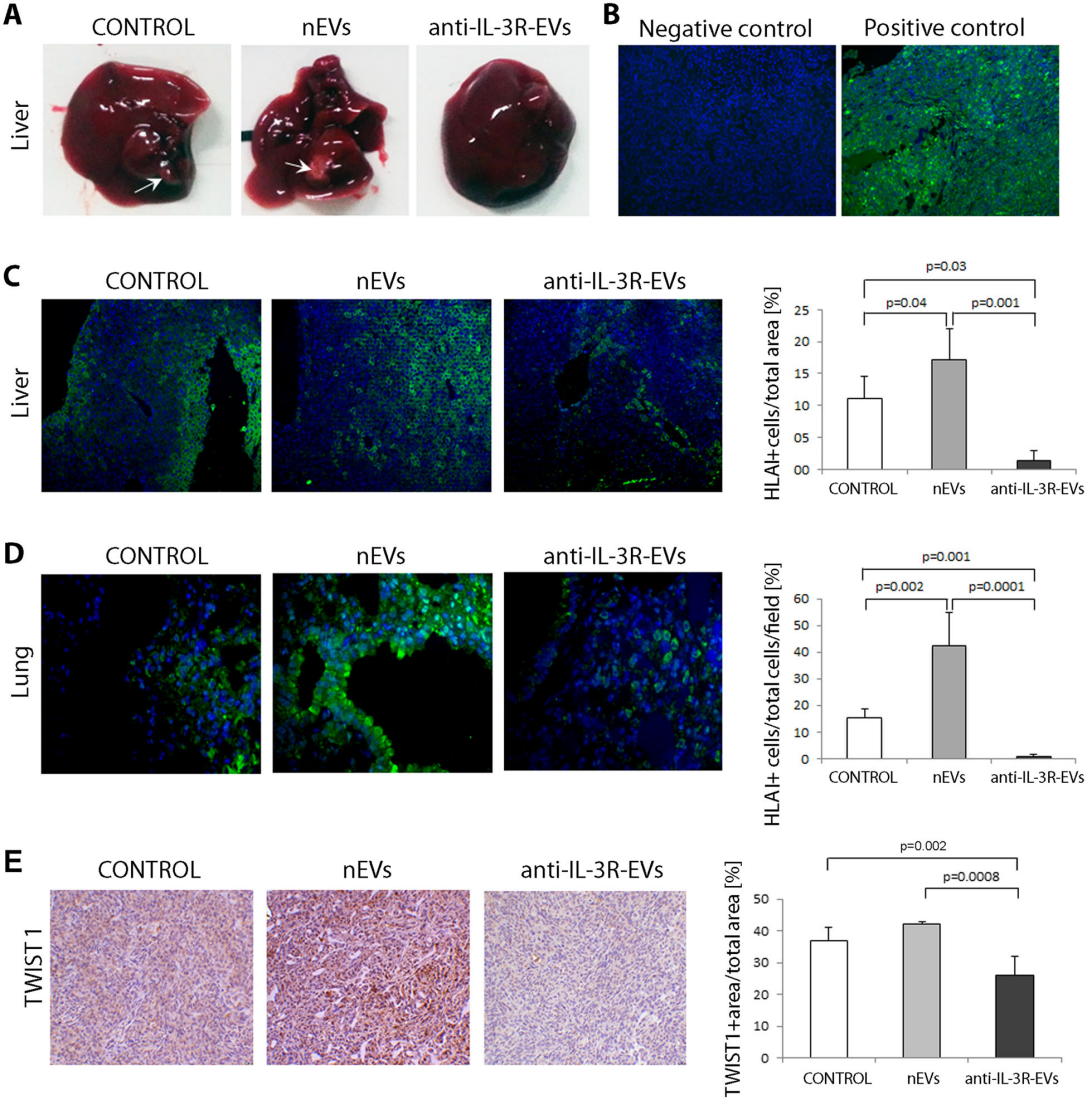
Effect of nEVs and anti-IL-3R-EVs on metastases generated from primary tumors. **a** Representative images of liver derived from primary tumors of mice that had been left untreated (CONTROL) or treated with TEC-EVs (nEVs and anti-IL-3R-EVs) (*n* = 4). **b** Representative images of negative (murine liver) and positive (primary tumors) immunofluorescence using an anti-HLA I antibody. Original magnification 100×, scale bar: 100 µm. **c** Representative immunofluorescence images of liver derived from mice (primary MDA-MB-231 tumors) that had been left untreated (CONTROL) or treated with TEC-EVs (nEVs and anti-IL-3R-EVs) and stained with an anti-HLA I antibody. Data are expressed as the percentage of HLA I+ cells/total area ± SD (*n* = 4). Original magnification 200×, scale bar: 50 µm. **d** Representative immunofluorescence images of lung derived from mice (primary MDA-MB-231 tumors) that had been left untreated (CONTROL) or treated with TEC-EVs (nEVs and anti-IL-3R-EVs) and stained with an anti-HLA I antibody. Data are expressed as the percentage of HLA I+ cells/total cells/field ± SD (*n* = 4). Original magnification 400×, scale bar: 25 µm. **e** Representative immunohistochemical images of TWIST1-positive staining of MDA-MB-231-derived primary tumors from animals that had been left untreated (CONTROL) or treated with TEC-EVs (nEVs and anti-IL-3R-EVs). Quantification of TWIST1 corresponds to the percentage of TWIST1 + area/total area ± SD (*n* = 4). Original magnification 200×, scale bar: 50 µm.

Since the inhibition of the canonical Wnt/β-catenin pathway was reported as a relevant mechanism of anti-IL-3R-EV action^31^, β-catenin expression was evaluated. As shown in Fig. 3e, treatment with anti-IL-3R-EVs was associated with significant downregulation in β-catenin. Moreover, as with β-catenin, the downregulation of Vimentin was detected in tumors from animals treated with anti-IL-3R-EVs (Fig. 3f). It has been shown that the Wnt/β-catenin network correlates with high metastatic TNBC behavior^39,40^. Therefore, metastases generated from primary tumors were evaluated. Liver macroscopic evaluation, shown in Fig. 4a, demonstrated the presence of huge metastatic nodules in tumors from mice treated with saline and nEVs, but not with anti-IL-3R-EVs. To confirm these data, immunofluorescence analysis was performed on the liver and lung, using an anti-human HLA I antibody to identify human cells in the mouse tissues. Interestingly, mice treated with anti-IL-3R-EVs displayed a significantly reduced number of HLA I+ cells in the liver and lung compared to saline- and nEV-treated animals (Fig. 4b–d). In several pathological contexts, including cancer, phenotypic processes that drive migratory and invasive properties rely on the expression of specific transcriptional factors^20^, and TWIST1 has been recognized as one of the main regulators^41^. Accordingly, anti-IL-3R-EV treatment led to the downregulation of TWIST1, both in vitro (Supplementary Fig. S6A, B) and in tumor tissues (Fig. 4e). We failed to detect changes in the expression of SNAI1 and SNAI2 (data not shown).

### TEC-EVs depleted of miR-24-3p (antago-miR-24-3p-EVs) impair proliferation and migration of MDA-MB-231 cells and, as anti-IL-3-EVs, interfere with lung metastasis generated by intravenous injection of MDA-MB-231 cells

We have previously shown that the regression of TEC-derived vessels observed in mice subjected to anti-IL-3R-EVs can be recapitulated by EVs recovered from TEC transfected with antago-miR-24-3p^31^. A comparison of MIRNOMIC analyses of anti-IL-3R-EVs and antago-miR-24-3p-EVs demonstrated that antago-miR-24-3p-EVs carried a rearranged miR cargo that was still therapeutically effective and able to recapitulate the in vivo anti-IL-3R-EV effects^31^. We therefore sought to evaluate whether the same cargo could be effective in mediating anti-IL-3R-EV antitumor effects. To this end, MDA-MB-231 cells were first investigated for miR-24-3p expression upon treatment with either nEVs or anti-IL-3R-EVs. We found that anti-IL-3R-EVs were able to decrease miR-24-3p content, compared to nEVs (Supplementary Fig. S6A). Similar results were detected when antago-miR-24-3p-EVs, obtained by transfecting TEC with antago-miR-24-3p (Supplementary Fig. S7), were used (Supplementary Fig. S6A). Although no difference in miR-24-3p content was detected when control and nEV-treated cells were compared, an increased miR-24-3p/TWIST1 level was found in cells transfected with scramble EVs (Supplementary Fig. S6A). Cell transfection may explain such a difference.

One of our previous studies has demonstrated that two proteins of the β-catenin disruption complex were targeted by miR-24-3p in TEC^31^. These data and the in vivo results led us to evaluate β-catenin expression in TNBC cell lines treated with nEVs, anti-IL-3R-EVs, and antago-miR-24-3p-EVs. Unlike in tumor samples recovered from mice subjected to anti-IL-3R-EVs, we failed to demonstrate changes in β-catenin in vitro (data not shown). However, antago-miR-24-3p-EVs, like anti-IL-3R-EVs, were able to significantly reduce TWIST1 expression, tumor cell number, and migration, and increase the apoptotic rate in vitro (Supplementary Fig. S6C–E). Possibly, due to a rapid mRNA translation, a high level of TWIST1 protein was detected, even in control cells. The basal level of TWIST1 detected in MDA-MB-231 cells may explain the high SD noticed in our experimental conditions.

**Fig. 5:**
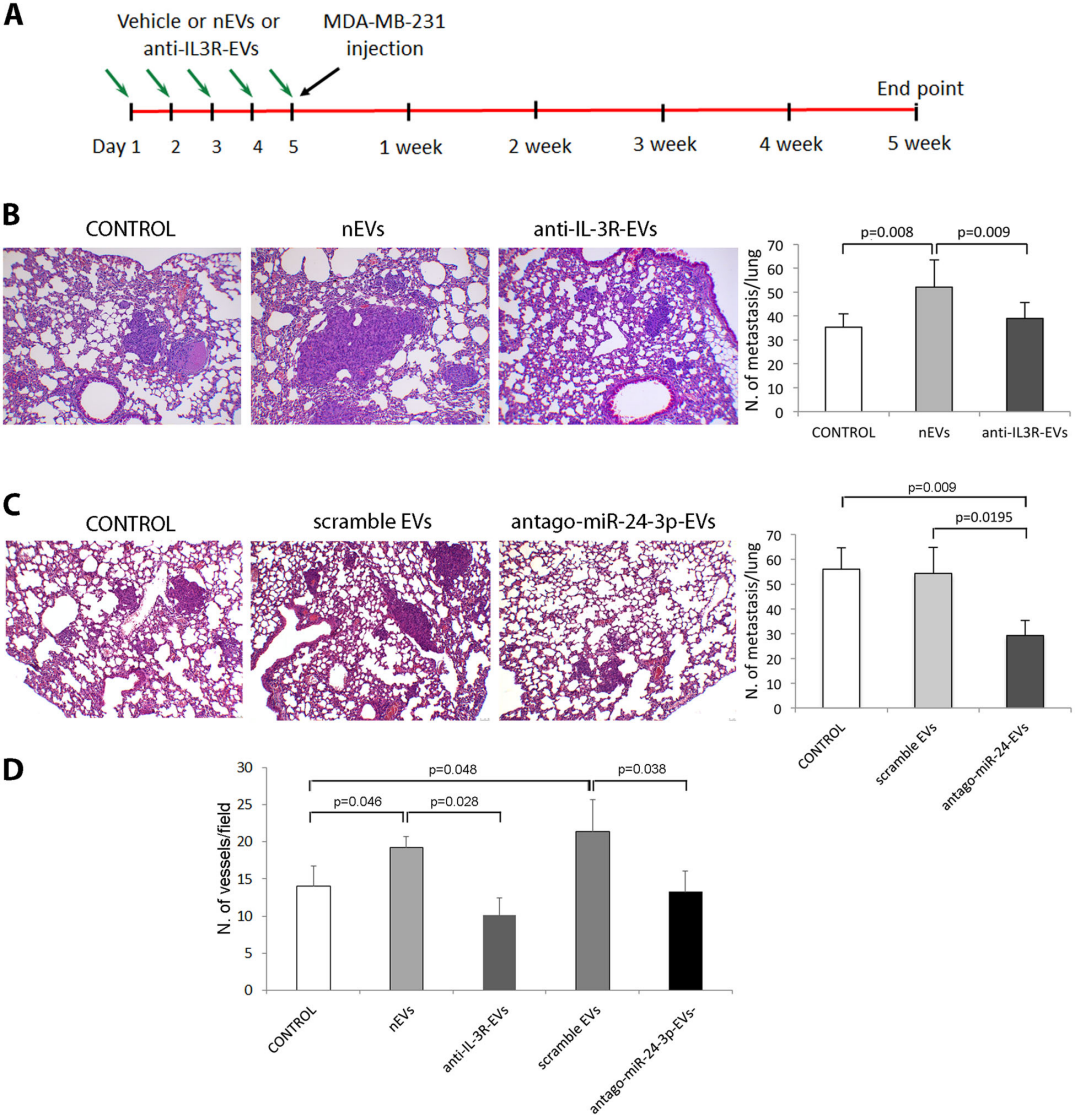
Anti-IL-3R-EV and antago-miR-24-3p-EV priming reduces lung metastasis formation. **a** Schematic representation of the experimental design to test the effect on metastasis formation of nEVs, anti-IL-3R-EV, and antago-miR-24-3p pretreatment before intravenous injection of tumor cells. **b** Representative hematoxylin and eosin images and quantification of lung metastasis derived from mice that had been left untreated (CONTROL) or treated with TEC-EVs (nEVs and anti-IL-3R-EVs) (*n* = 4). Original magnification 100×, scale bar: 100 µm. The number of metastases was expressed as the number of metastases/lung ± SD (*n* = 4). **c** Representative hematoxylin and eosin images and quantification of lung metastases derived from mice that had been left untreated (CONTROL) or treated with scrambled EVs and antago-miR-24-3p-EVs (*n* = 4). Original magnification 100×, scale bar: 100 µm. The number of metastases was expressed as the number of metastases/lung ± SD (*n* = 4). **d** Evaluation of vessel density in lungs derived from mice that had been left untreated (CONTROL) or treated with nEVs and anti-IL-3R-EVs and with scramble EVs and antago-miR-24-3p-EVs. Data are expressed as number of vessels per field ± SD (*n* = 4).

Hence, since nEVs were able to promote the metastases generated from primary tumors, we first sought to determine whether circulating nEVs can also contribute to lung metastasis formation of intravenously injected MDA-MB-231 cells. The effect of nEVs was compared to that of anti-IL-3R-EVs. To address this issue, either nEVs or anti-IL-3R-EVs were injected intravenously for 5 consecutive days. On day 5, MDA-MB-231 cells were injected intravenously, and the animals were followed for 5 weeks (Fig. 5a). As shown in Fig. 5b, lung metastasis formation increased in mice treated with nEVs. Interestingly, this effect was significantly reduced by anti-IL-3R-EV treatment. We therefore investigated whether antago-miR-24-3p-EVs could recapitulate anti-IL-3R-EV-mediated protection against lung metastasis formation. As shown in Fig. 5c, antago-miR-24-3p-EVs were as effective as anti-IL-3R-EVs in reducing lung metastasis formation. Saline and scramble miR served as controls. To evaluate whether vascularization could contribute to these results, the whole lung vessel area was evaluated in mice primed with nEVs, anti-IL-3R-EVs, or antago-miR-24-3p-EVs. Indeed, a significantly reduced number of the lung vessels was found in the mice primed with anti-IL-3R-EVs or antago-miR-24-3p-EVs (Fig. 5d), indicating that circulating TEC-EVs may provide the soil for cancer cell homing possibly due to their pro-angiogenic properties.

### SPRY2 undergoes upregulation in response to anti-IL-3R-EV and antago-miR-24-3p-EV challenge

**Fig. 6:**
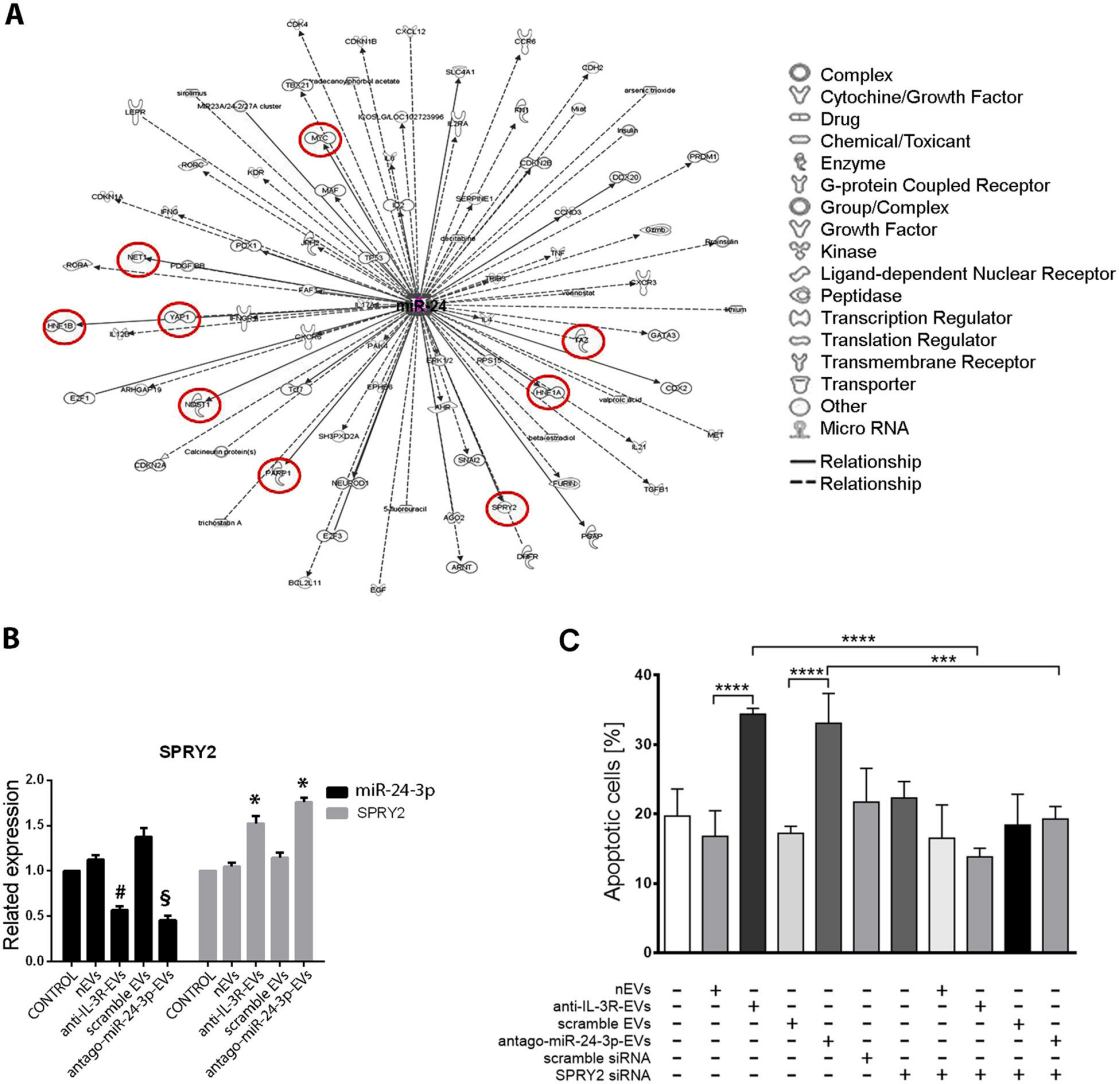
miR-24-3p gene network identified using Ingenuity Pathway Analysis (IPA). **a** IPA predicted target genes for miR-24-3p. Arrowheads represent activating relationships, whereas solid or dotted edges indicate direct and indirect relationships, respectively. **b** qRT-PCR for SPRY2 in unstimulated MDA-MB-231 cells (CONTROL) or upon nEV, anti-IL-3R-EV, scramble EV, and antago-miRNA-24-3p-EV treatment. Related expression of miR-24-3p for each condition is reported. ^#^*p* < 0.05 anti-IL-3R-EVs vs. nEVs; ^§^*p* < 0.05 antago-miR-24-3p-EVs vs. scramble EVs; *p < 0.05 anti-IL-3R-EVs vs. nEVs, and antago-miR-24-3p-EVs vs. scramble. **c** The apoptotic rate was evaluated after 24 h of treatment of MDA-MB-231 cells untransfected or transfected with scramble or SPRY2 siRNA. Saline or TEC-EVs (nEVs, anti-IL-3R-EVs, scramble EVs, and antago-miR-24-3p-EVs) were used. Data are expressed as the percentage of apoptotic cells ± SD (*n* = 6). ***p < 0.005: antago-miR-24-3p-EVs vs. SPRY2-silenced cells + antago-miR-24-3p-EVs; ****p < 0.0001: nEVs vs. anti-IL-3R-EVs, scramble EVs vs. antago-miR-24-3p-EVs, and anti-IL-3R-EVs vs. SPRY2-silenced cells + anti-IL-3R-EVs.

To gain further insight into anti-IL-3R-EV and antago-miR-24-3p-EV mechanisms of action, an integrated miR-24-3p interaction network was performed. The network that was predicted by ingenuity pathway analysis (IPA) for miR-24-3p target genes identified several genes (Fig. 6a). Some genes with a direct relationship with miR-24-3p, such as HNF1A, HNF1B, SPRY2, TAZ, YAP, C-MYC, NET1, PARP1, and NDST1, were therefore evaluated in MDA-MB-231 cells treated with anti-IL-3R-EVs and antago-miR-24-3p-EVs. As shown in Fig. 6b and Supplementary Fig. S8, only SPRY2 was significantly upregulated upon anti-IL-3R-EV and antago-miR-24-3p-EV treatment. These data suggest that upregulation of SPRY2 may contribute to either anti-IL-3R-EVs or antago-miR-24-3p-EV mechanism of action. Indeed, we found that SPRY2 silencing inhibits anti-IL-3R-EV- and miR-24-3p-EV-mediated apoptosis (Fig. 6c, Supplementary Fig. S9).

## Discussion

TNBC is an aggressive highly metastatic breast cancer subtype lacking estrogen, progesterone, and HER-2 receptors^42^ and proven target therapies^43^. The identification of molecular markers/effective therapeutics that impact upon tumor progression is therefore a future clinical challenge.

EVs derived from different TME components, including TEC, influence key aspects of cancer growth/progression, and have recently been recognized as being of great importance for tumor targeting^44^. The goal of this study was to investigate whether IL-3Rα blockade on TEC impacts tumor progression via EVs. A previous study demonstrated that IL-3Rα blockade on TEC, by an anti-IL-3Rα^36^, changed the miR-EV cargo and had striking effects on tumor vessel formation^31^, suggesting that reprogramming TEC-EVs may also be instrumental in tumor targeting. To provide the rationale for TEC targeting in TNBC, IL-3Rα expression was evaluated in human TNBC samples. We demonstrated that tumor cells highly expressed IL-3Rα in 55.5% of the TNBC examined. Of note, TNBC cell lines, but not MCF10A cells, also expressed IL-3Rα. How the IL-3Rα and its ligand, IL-3, take part in TNBC cell biology is still to be established, and further studies are required to address this issue. However, our data suggest that TNBC may serve as a valuable model with which to investigate the impact of IL-3Rα targeting on cell-to-cell communication in TME, as IL-3Rα was highly expressed in TEC.

We have demonstrated herein that nEV treatment boosts cancer cell viability and migration, while anti-IL-3R-EVs significantly impair both, and induce apoptosis in vitro. E- and N-cadherin expression in cells treated with anti-IL-3R-EVs further sustains their biological activity. EV composition differs significantly in untransformed and transformed cells and accounts for the different biological actions^24^. Accordingly, EVs from normal endothelial cells have no effect on tumor cell growth/migration/apoptosis, whether they were unstimulated or stimulated with IL-3, indicating that the effects of nEVs strictly rely on the unique TEC phenotype.

Intercellular crosstalk can occur via secreted mediators and EVs in the TME^45^. Indeed, tumor-derived EVs and EVs from the TME impact upon tumor progression also by promoting angiogenesis^24^. Furthermore, vessel density in primary tumors correlates with metastasis^46^. We herein demonstrated that, unlike nEVs, anti-IL-3R-EVs inhibit tumor neovascularization without significantly impacting VM^21^. Proteins, mRNAs, and miRs shuttled within TME-derived EVs largely provide services to the tumor^47^. In fact, a previous study of ours demonstrated that miR-214-3p and miR-24-3p, which target the canonical wingless Wnt/β-catenin pathway, were differentially regulated in nEVs and anti-IL-3R-EVs, and mediate their pro- and antiangiogenic effects, respectively^31^. In this study, we have demonstrated that anti-IL-3R-EVs also reduce β-catenin expression in tumor-bearing mice, suggesting that anti-IL-3R-EVs are also able to target the canonical wingless Wnt/β-catenin pathway in neoplastic cells. β-catenin accumulation and the acquisition of mesenchymal markers, as Vimentin, in tumor cells, are associated with cancer cells’ ability to spread to distant sites^48–50^. Indeed, anti-IL-3R-EVs were found effective in reducing the expression of Vimentin, and animals treated with anti-IL-3R-EVs were almost protected from the occurrence of liver and lung metastasis. A harmonized set of transcriptional factors drives the activation of the metastatic program^20^, and TWIST1 is one of them^41^. TWIST1 belongs to a family of transcriptional factors highly expressed in most cancers, and particularly in those highly metastatic^20,41^. We demonstrated that TWIST1 was reduced in vitro and more importantly, in mice treated with anti-IL-3R-EVs. TWIST1 expression is strictly controlled at transcriptional and post-transcriptional levels^20^. Several different miRs have been shown to regulate TWIST1 at the post-transcriptional level^20^. Herein, we have demonstrated that, as anti-IL-3R-EVs, EVs depleted of miR-24-3p reduced TWIST1 expression in stimulated cells. miR-214-3p, which was also found enriched in anti-IL-3R-EVs and miR-24-3p-EVs^31^, has been involved in TWIST1 post-transcriptional regulation in ovarian cancers^51^. Unfortunately, we failed to detect changes in TWIST1 expression when nEVs enriched in miR-214-3p were used to stimulate MDA-MB-231 and MD-MB-453 cells (data not shown). Vimentin, TWIST1, and β-catenin have been linked through STAT3^52^, and more recently, the role of miR-551b-3p in controlling STAT3 transcription and TNBC progression has been documented^53^. Again, we failed to detect miR-551b-3p among miRs differentially expressed in nEVs and anti-IL-3R-EVs^31^ and differences in STAT3 expression/activation in our model (data not shown). This suggests that the anti-IL-3R-EV- and antago-miR-24-3p-EV-mediated downregulation of TWIST1 as well as their biological activities may rely on the combined action of a pattern of shared miRs, we have previously described^31^. However, as EVs also induce their biological effects by transferring lipids, proteins, mRNAs, and transcription factors^47,49^, it might be necessary to consider the entire EV cargo to explain the anti-IL-3R-EV and miR-24-3p-EV mechanism of action.

Although chemotherapy is still the main modality for TNBC treatment, the recurrence of metastasis hampers the improvement of patient outcomes^54,55^. The development of novel therapeutic options to improve TNBC patient survival is therefore a concrete clinical need. The results in primary tumors and the ability of antago-miR-24-3p-EVs to recapitulate anti-IL-3R-EV action in vitro led us to determine the impact of circulating anti-IL-3R-EVs/antago-miR-24-3p-EVs in preventing the formation of lung metastasis generated by tumor cell intravenous injection. Indeed, we demonstrated that lung metastasis formation was reduced in mice that had been primed with both anti-IL-3R-EVs and antago-miR-24-3p-EVs. EVs released by cancer stem cells were found to be instrumental for premetastatic niche formation^56^. We herein demonstrate that nEVs are also instrumental for metastasis formation, while anti-IL-3R-EVs and antago-miR-24-3p-EVs were therapeutically effective in reducing their formation. The possibility that this effect relied on their pro-angiogenic/antiangiogenic properties is sustained by the increased/reduced vascular network in the lung of animals primed with nEVs or anti-IL-3R-EVs and antago-miR-24-3p-EVs, respectively.

To gain insight into the potential signaling involved in the anti-IL-3R-EV and antago-miR-24-3p-EV mechanisms of action, IPA was interrogated to identify miR-24-3p-interacting genes. Of the most significant miR-24-3p interactors evaluated, only SPRY2 was found to be upregulated upon anti-IL-3R-EV and antago-miR-24-3p-EV challenge. SPRY2, which belongs to the sprouty gene family, acts as a negative regulator of several receptor tyrosine kinases that are also involved in angiogenesis^57^. Moreover, the expression of the SPRY2 gene was found to be repressed in breast cancers^58^. Accordingly, we found that SPRY2 was downregulated upon nEV treatment, while anti-IL-3R-EVs and antago-miR-24-3p-EVs rescued SPRY2 expression. Moreover, we found that SPRY2 silencing prevented anti-IL-3R-EV- and antago-miR-24-3p-EV-mediated apoptosis.

Overall, this study demonstrates that IL-3Rα blockade on TEC reprograms EVs, which then acquire the ability to change the expression of Vimentin, β-catenin, and TWIST1, and reduce angiogenesis and the metastatic spread of primary tumors. Moreover, anti-IL-3R-EV priming was found to be therapeutically effective in reducing lung metastasis, possibly due to its antiangiogenic properties and/or interference with cancer cell homing. Moreover, we provide the first evidence that inflammatory cells, TEC, and, more importantly, tumor cells, in human TNBC samples, express IL-3Rα. Finally, since EVs released upon TEC targeting can be considered the leading effectors of IL-3Rα blockade, the results of the present study provide evidence for the therapeutic effectiveness of this antibody-based targeted approach in TNBC.

## Materials and methods

Detailed information in this section is reported in Supplementary Information.

### Immunohistochemistry and immunofluorescence on human and animal samples

A series of 27 patients diagnosed with TNBC between 2011 and 2012 was retrieved from the files of the Pathology Department of the Città della Salute e della Scienza Hospital (Turin). The study was conducted in accordance with the guidelines and regulations defined by the Research Ethics Committee for human Biospecimen Utilization (Department of Medical Sciences—ChBU) of the University of Turin. Representative blocks were obtained as previously described^59^. Immunohistochemistry was performed using an automated slide-processing platform (Ventana BenchMark AutoStainer, Ventana Medical Systems, Tucson, AZ, USA), with Universal DAB Detection Kit detection systems. In all, 5-µm paraffin-embedded tumor sections were stained with CD31 and PAS to quantify CD31+ vessels and VM expressed as CD31−/PAS+ vessels. Masson’s trichrome staining was also used. Ten sections/tumors were analyzed using ImageJ software, and the results were expressed as the number of CD31+/PAS+/fields ± SD. Moreover, tumor sections were analyzed using the ApopTag^®^Plus Peroxidase In Situ Apoptotic Detection kit (Millipore, #S7101). Immunohistochemistry for the detection of Vimentin, TWIST1, and β-catenin was performed using a monoclonal anti-Vimentin antibody (Sigma #V5255), a polyclonal anti-TWIST1 antibody (Abcam #ab49254), and a polyclonal anti-β-catenin antibody (Abcam #ab16051). Quantifications of Vimentin- and TWIST1-positive area were performed using Fiji software^60^. The analysis of β-catenin-positive cells was performed by two independent pathologists and expressed as Quick score (Q)^61^. MDA-MB-231 cells were detected in livers and lungs by immunofluorescence using anti-HLA I (Santa Cruz Biotechnology, #sc-25619). Details are reported in Supplementary Information.

### Cell cultures

The MDA-MB-231, MDA-MB-453, and MCF10A cell lines were purchased from ATCC.

Human-derived TEC was obtained from surgical tumor specimens using anti-CD105-positive selection^62^. Cells were cultured as described previously^11,62^.

Primary human umbilical vein endothelial cells, purchased from ATCC and used as controls of nontumoral endothelial cells, were untreated or treated with IL-3 (10 ng/ml) to obtain EVs (EV ctr and EV IL-3, respectively), as previously described^63^.

### EV isolation and characterization

In selected experiments, starved TEC was cultured for 24 h in the presence of 1 μg/ml Human IL-3Rα/CD123 MAb (R&D Systems, #MAB301-100, Clone 32703). Untreated TEC served as controls. For EV isolation, TEC, untreated or pretreated by blocking IL-3Rα, was cultured for 24 h in fetal bovine serum (FBS)-free EndoGro medium. The conditioned medium was centrifuged for 30 min at 3000 *g* to remove cell debris and apoptotic bodies, and then submitted to microfiltration with 0.22-μm filters (MF-Millipore^™^) to remove larger vesicles. The TEC-EV suspension was then stored at −80 °C until further use. In specific experiments, TEC was transfected with antago-miR-24-3p (Ambion, cat #4464085, assay ID MH1073) or scramble siRNA (Ambion, cat #4464077). Details are reported in Supplementary Information.

### EV characterization

EVs were analyzed using NTA, electron microscopy, and FACS analysis. Moreover, EV flow cytometry analysis was performed using the MACSPlex Exosome Kit (human, Miltenyi Biotec), following the manufacturer’s protocol^64^. The CD63 exosomal marker was also analyzed by western blot. Details are reported in Supplementary Information.

### Cell counting, apoptosis, scratch test, and SPRY2 silencing

Apoptosis assay: cells seeded in 6-well plates were stimulated for 24 h with different types of TEC-EVs (nEVs, anti-IL-3R-EVs, scramble EVs, and antago-miR-24-3p-EVs) (2 × 10^8^ EVs/ml) in FBS-free DMEM. The effective dose was selected with reference to the preliminary results obtained using different EV concentrations (data not shown). Treated and untreated cells were analyzed using Muse^®^ Annexin V & Dead Cell Kit (Millipore, #MCH100105). Cell proliferation was assayed by direct cell count by two different operators. Scratch assay: cells seeded in 24-well plates and grown until confluence in DMEM 10% FBS were stimulated with TEC-EVs (as above) in DMEM FBS-free medium and analyzed 24 h later. The results were expressed as mean distance (0–24 h) ± SD. In selected experiments, SPRY2 was silenced in MDA-MB-231 cells by transfecting siRNA scramble (Qiagen, Cat No. 1027310) or siRNA for SPRY2 (Qiagen, Cat No. SI00081788) using HiPerFect Transfection Reagent (Qiagen, Cat No. 301704) (Supplementary Information).

### Sphere-formation assay

To test the ability of MDA-MB-231 cells to grow in nonadhesive conditions as floating spheres, cells were plated in 6-well nonadherent plates, at a concentration 50 × 10^3^/well, in 2 ml of sphere-formation medium in the presence of nEVs or anti-IL-3R-EVs (1×10^8^ EVs/ml). Data are expressed as the number of sphere/sample ± SD (Supplementary Information).

### Tumor growth and model of metastasis formation in vivo

Animal studies were conducted in accordance with the Italian National Institute of Health Guide for the Care and Use of Laboratory Animals (protocol No. 944/2015-PR). Mice were housed according to the guidelines of the Federation of European Laboratory Animal Science Association and the Ethical Committee of the University of Turin. The investigators (at least 2) were blinded when assessing the outcome. Tumors were obtained by injecting MDA-MB-231 cells in Matrigel into the mammary fat pad of SCID mice (8 weeks/female) (4 mice/group) (1 × 10^6^ cells per injection). After 3 weeks, when tumors became palpable, animals were treated with saline, nEVs, or anti-IL-3R-EVs (1 × 10^10^ EV/tumor) twice a week for 3 additional weeks (Fig. 3a). At day 45, tumors were embedded in paraffin (*n* = 4/each condition). To evaluate metastasis formation after intravenous tumor injection, EVs (1 × 10^10^ EV/injection) were intravenously injected for 5 days into SCID mice (Fig. 5a). On day 5, 0.6 × 10^6^ MDA-MB-231 cells were injected intravenously. The mice were sacrificed after 5 weeks and lungs analyzed. Lung metastases were counted using ImageJ in five nonsequential sections. The results were expressed as mean ± SD of metastasis per lung (*n* = 4/each condition)^56^. Lung vessels with red blood cells inside were quantified in lung sections stained with Masson’s trichrome and expressed as the number of vessels/field ± SD. Details are reported in Supplementary Information.

### Real-time PCR

Real-time polymerase chain reaction (PCR) was performed to detect miR-24-3p, SPRY2, and TWIST1 in TEC-EVs and MDA-MB-231 cells as indicated. Total RNA from TEC-EV samples and MDA-MB-231 cells was extracted using the RNAeasy kit (Qiagen). RNA was reverse-transcribed using miScript II RT Kit (Qiagen).

### Western blot

Western blot was performed as previously described^31^ and reported in Supplementary Information.

### miR-24-3p target validation

Ingenuity pathway analysis (IPA) was used to predict the target genes for miR-24-3p. The miR Target Filter tool was set up on IPA (Qiagen: http://www.qiagenbioinformatics.com/products/ingenuity-pathway-analysis/) to associate miR-24-3p with predicted mRNA targets. miR-24-3p target expression (HNF1B, TAZ, PARP1, HNF1A, SPRY2, MYC, YAP, NET1, and NDST1) was evaluated by RT-PCR using actin-β as the housekeeping transcript. Primer sequences and details are in Supplementary Table S2

### Statistical analysis

All data are reported as mean ± SD. Comparison between two groups was carried out by *t* test. Our data passed normality and equal-variance tests. Comparisons among ≥3 were performed by one- way ANOVA followed by Tukey’s multiple-comparison test. The cutoff for statistical significance was set at *p* < 0.05. All in vitro or in vivo results are representative of at least 3 independent experiments. All statistical analyses were carried out on Graph Pad Prism version 5.04 (Graph Pad Software, Inc., USA).

## Supplementary information

Supplemental Material

authorship form

email authorship confirmation
