# Correction: Targeting IL-3Rα on tumor-derived endothelial cells blunts metastatic spread of triple-negative breast cancer via extracellular vesicle reprogramming

**DOI:** 10.1038/s41389-023-00483-1

**Published:** 2023-07-25

**Authors:** Tatiana Lopatina, Cristina Grange, Claudia Cavallari, Victor Navarro-Tableros, Giusy Lombardo, Arturo Rosso, Massimo Cedrino, Margherita Alba Carlotta Pomatto, Malvina Koni, Francesca Veneziano, Isabella Castellano, Giovanni Camussi, Maria Felice Brizzi

**Affiliations:** 1 Department of Medical Sciences, University of Turin, Turin, Italy; 2 2i3T Scarl University of Turin, Turin, Italy

**Keywords:** Breast cancer, Drug development

Correction to: *Oncogenesis* 10.1038/s41389-020-00274-y published online 10 October 2020

Following the publication of this article an error was noted in figure assembly for Figure 2E, where an image from the control group had been mistakenly included to represent EV treatment.

The correct figure showing EV treatment is presented below.
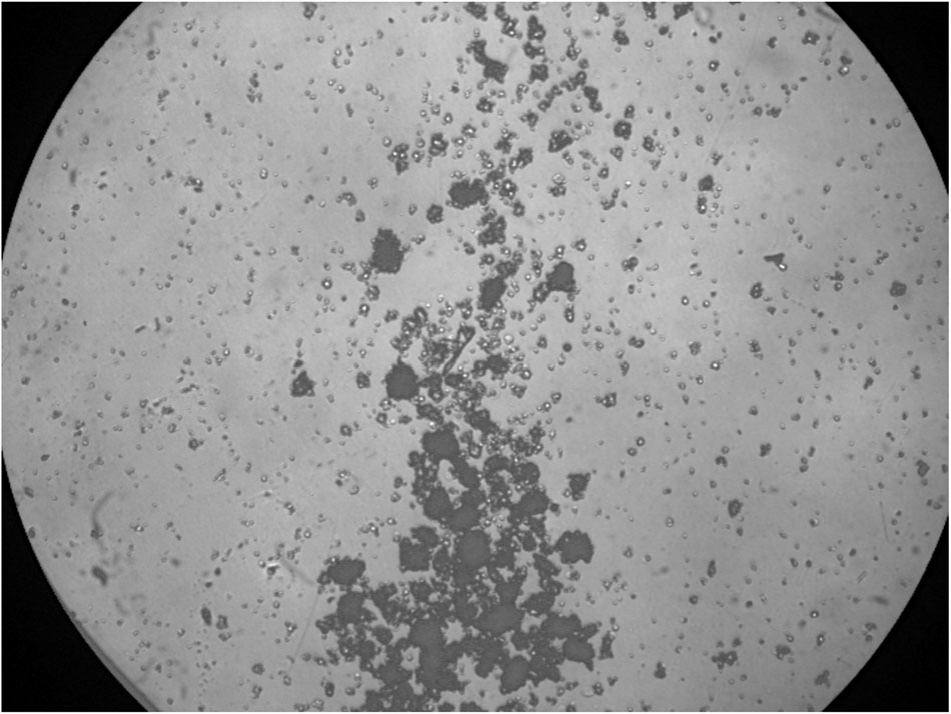


The original article has been corrected.

