# Supplementary material for: Targeting IL-3Rα on tumor-derived endothelial cells blunts metastatic spread of triple-negative breast cancer via extracellular vesicle reprogramming
Source: Oncogenesis. 2020 Oct 10;9(10):90. doi: 10.1038/s41389-020-00274-y (PMC7548009; doi:10.1038/s41389-020-00274-y)
Supplement: Supplementary file 1 — Supplemental Material [file 41389_2020_274_MOESM1_ESM.docx]

**Targeting IL-3Rα on tumor-derived endothelial cells blunts metastatic spread of triple negative breast cancer via extracellular vesicle reprogramming**

Tatiana Lopatina*^1^*, Cristina Grange*^1^*, Claudia Cavallari*^2^*, Victor Navarro-Tableros*^2^*, Giusy Lombardo*^1^*, Arturo Rosso*^1^*, Massimo Cedrino*^2^*, Margherita Alba Carlotta Pomatto*^1^*, Malvina Koni^1^, Francesca Veneziano*^1^*, Isabella Castellano*^1^*, Giovanni Camussi*^1^**, and Maria Felice Brizzi*^1^**

***^1^****Department of Medical Sciences, University of Turin, Turin, Italy,* ***^2^****2i3T Scarl University of Turin,*

**TL and CG contributed equally**

**Running title:** IL-3Rα blockade on TEC blunts TNBC dissemination

**Supplementary Data**

### **Supplementary Fig. S1**

**
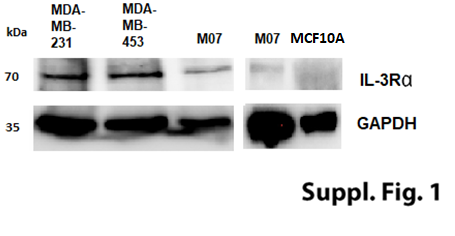
**

**Supplementary Fig. S1. IL-3Rα expression by MDA-MB-453 and MDA-MB-231 but not by MCF10A cells.** Representative western blot showing the presence of IL-3Rα in MDA-MB-231 and MDA-MB-453, but not in MCF10A cells. MO7 cells served as positive control and GAPDH as housekeeping.

**Supplementary Fig. S2**


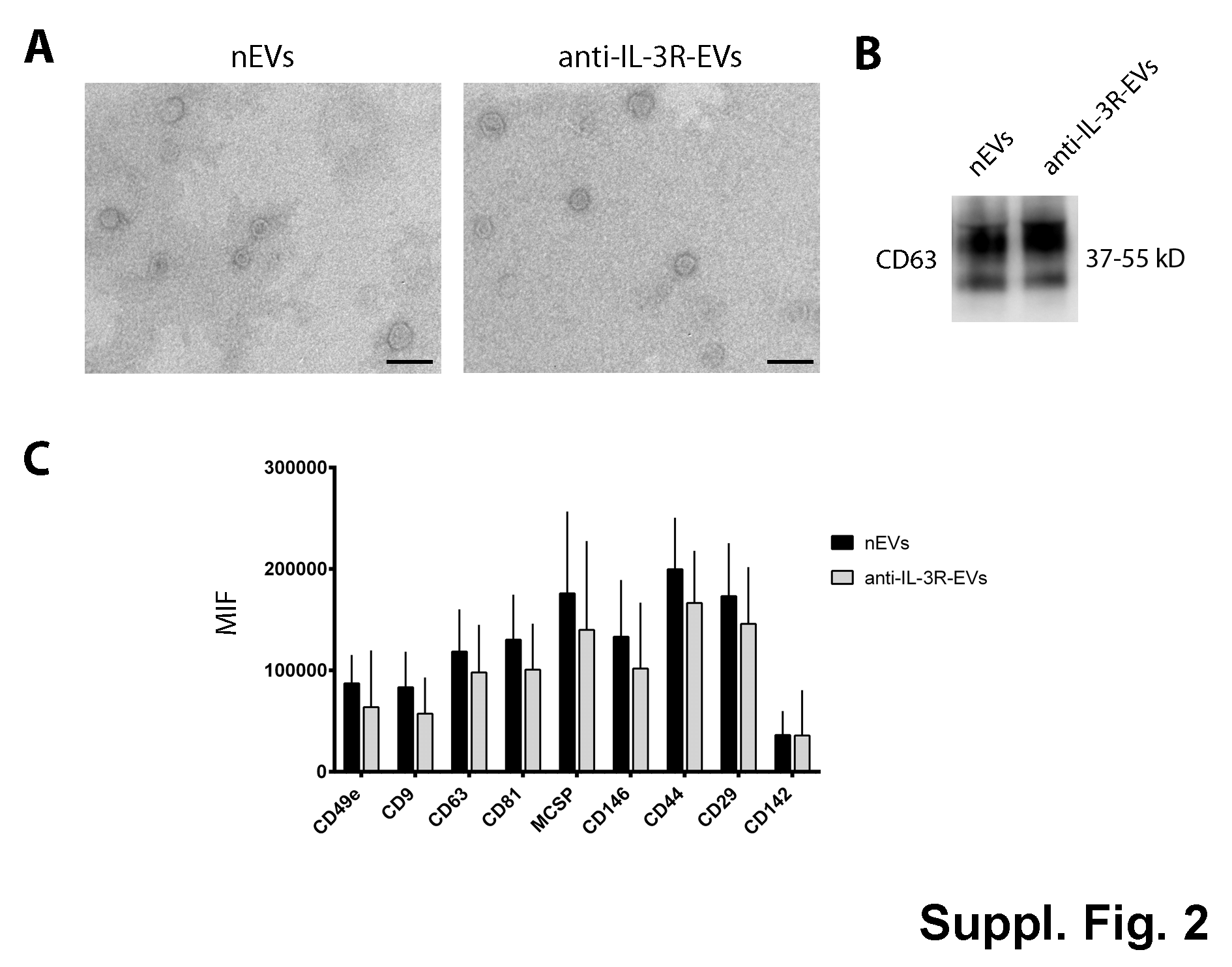


**Supplementary Fig. S2. TEC-EV characterization**. **a** Representative TEM micrographs of nEVs and anti-IL-3R-EVs showing no difference in size and morphology. Original magnification 140K, scale bar: 100µm. **b** Representative western blot of CD63 on nEVs and anti-IL-3R-EVs. **c** FACS analysis expressed as median APC fluorescence intensity (MFI) of surface markers on nEVs and anti-IL-3R-EVs using the MACSPlex Exosome Kit. Data are expressed as mean ±SD (n=5 different EV batches for nEVs and anti-IL-3R-EVs).

**Supplementary Fig. S3**

**
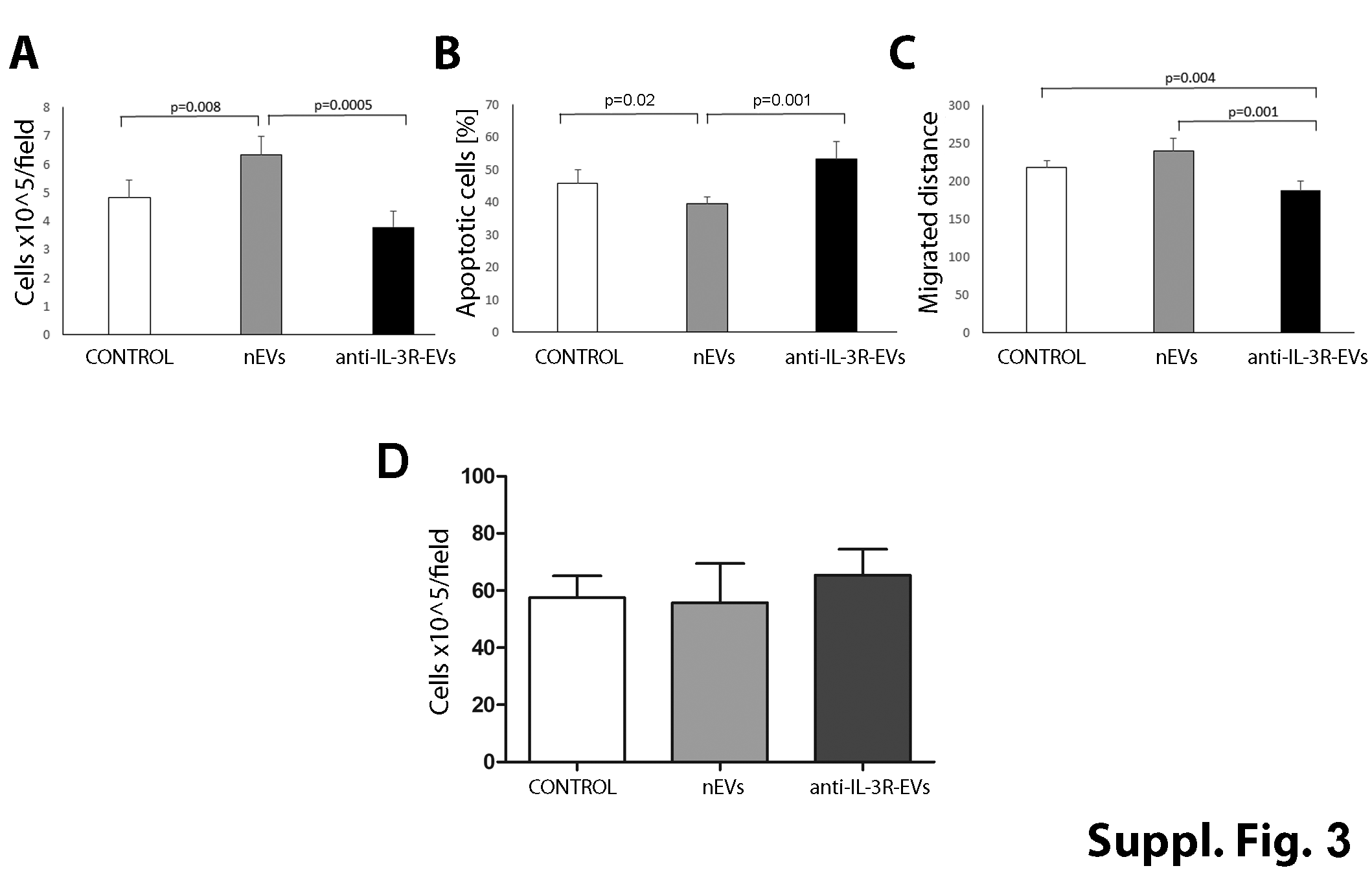
**

**Supplementary Fig. S3. *In vitro* effects of nEVs and anti-IL-3-EVs on MDA-MB-453 cells and MCF10A cells.**

**A** MDA-MB-453 cells were stimulated for 48h with nEVs or anti-IL-3R-EVs (data are expressed as mean ±SD) (n=4). **B** MDA-MB-453 cells were stimulated with nEVs and anti-IL-3R-EVs for 24h (data are expressed as percentage of apoptotic cells ±SD) (n=4). **C** Migration assay of cells stimulated with nEVs and anti-IL-3R-EVs for 24h Data are expressed as distance ±SD (n=4). **D** MCF10A cells were stimulated for 48h with nEVs or anti-IL-3R-EVs (data are expressed as mean ±SD) (n=4).

**Supplementary Fig. S4**


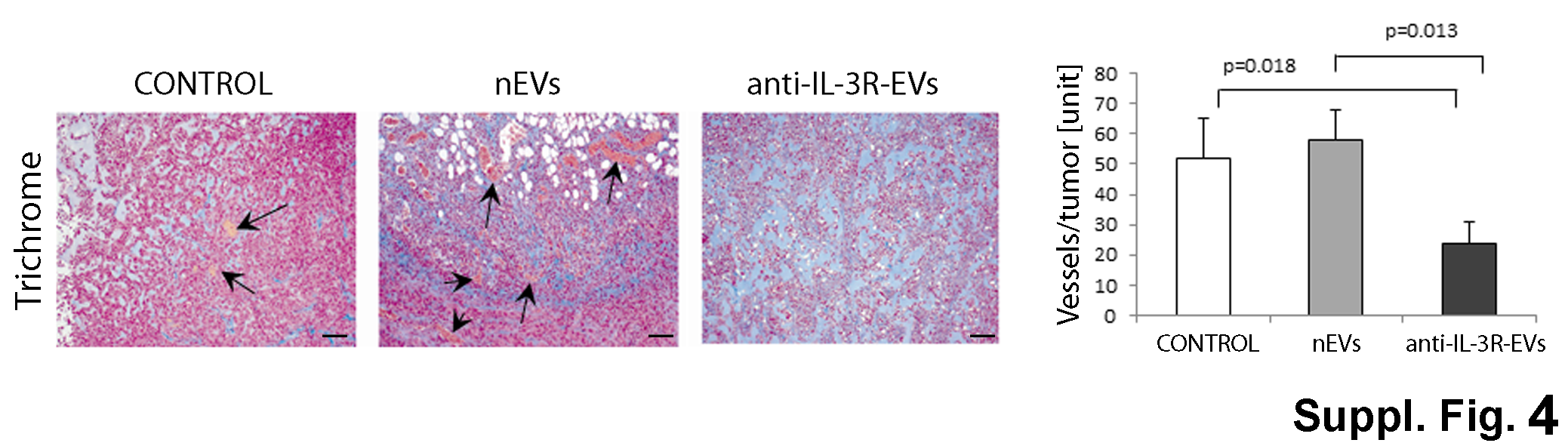


**Supplementary Fig. S4. Effects of nEV and anti-IL-3R-EV treatment on vasculature.** Representative images of tumors untreated (CONTROL) or treated with TEC-EVs (nEVs and anti-IL-3R-EVs) stained using Masson’s trichromic reaction. Vessels within MDA-MB-231 tumors were expressed as vessel/fields ±SD (n=4). Original magnification 200X, scale bar: 50µm.

**Supplementary Fig. S5**


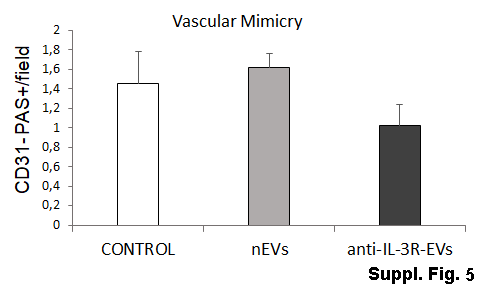


**Supplementary Fig. S5. Effects of nEV and anti-IL-3R-EV treatment on vasculogenic mimicry.** Quantification of CD31-/PAS+ vessels of tumors untreated (CONTROL) or treated with TEC-EVs (nEVs and anti-IL-3R-EVs). Results are expressed as vessel CD31-/PAS+ per field ±SD (n=4).

**Supplementary Fig. S6**


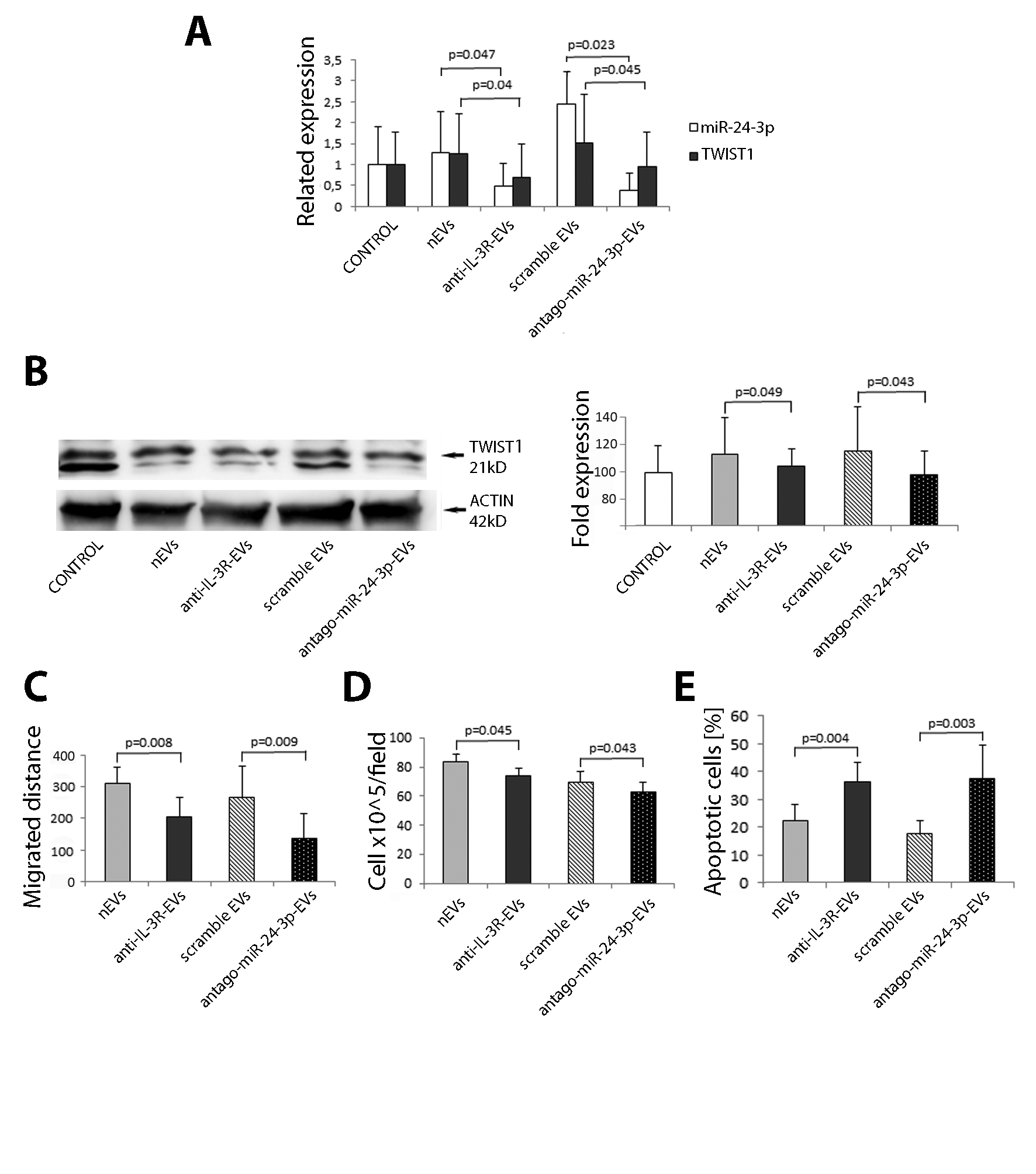


**Supplementary Fig. S6. *In vitro* effects of antago-miR-24-3p-EVs on MDA-MB-231 cells. a** Related expression of miR-24-3p and TWIST1 (RT-PCR) on MDA-MB-231 cells untreated (CONTROL) or treated with nEVs, anti-IL-3R-EVs, scramble EVs and antago-miR-24-3p-EVs (n=6). **b** Representative image of western blot and quantification of TWIST1 expression in MDA-MB-231 cells which have been untreated (CONTROL) or treated with nEVs, anti-IL-3R-EVs, scramble EVs and antago-miR-24-3p-EVs. Data are expressed as the mean ±SD normalized to Actin (n=3). **c** Cells were stimulated for 48h with nEVs, anti-IL-3R-EVs, scramble EVs, and antago-miR-24-3p-EVs (data are expressed as mean ±SD (n=6). **d** Quantification of MDA-MB-231 cell migration expressed as migrated distance ±SD after nEV, anti-IL-3R-EV, scramble EV and antago-miR-24-3p-EV stimulation (n=6). **e** Quantification of cell apoptosis expressed as percentage of apoptotic cells ±SD in MDA-MB-231 cells which have been treated with nEVs, anti-IL-3R-EVs, scramble EVs and antago-miR-24-3p-EVs (n=6).

**Supplementary Fig. S7**


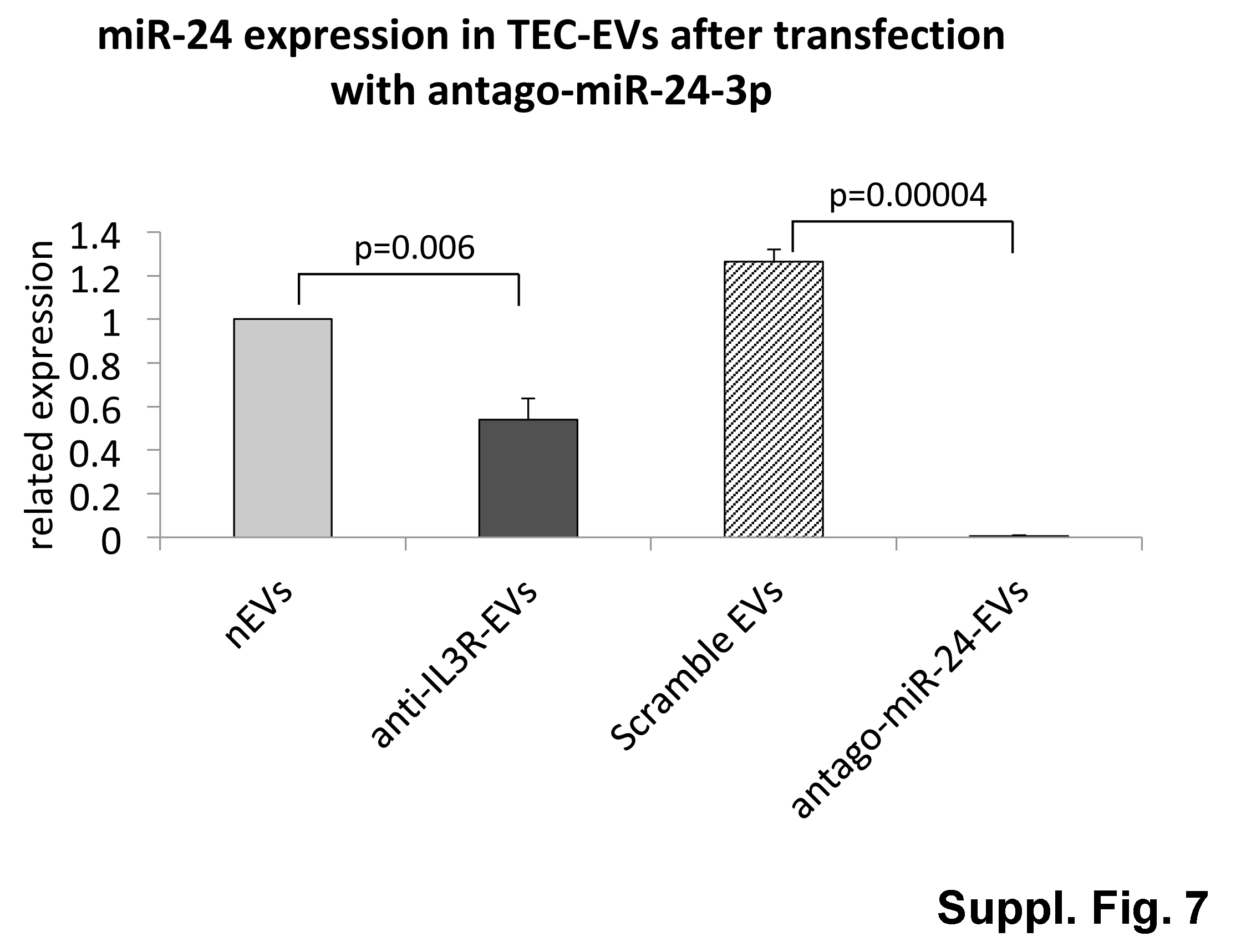


**Supplementary Fig. S7. miR-24-3p expression in TEC-EVs after transfection with** **antago-miR-24-3p.** miR-24-3p expression in TEC-EVs after transfection with antago-miR-24-3p (n=6).

**Supplementary Fig. S8**

**
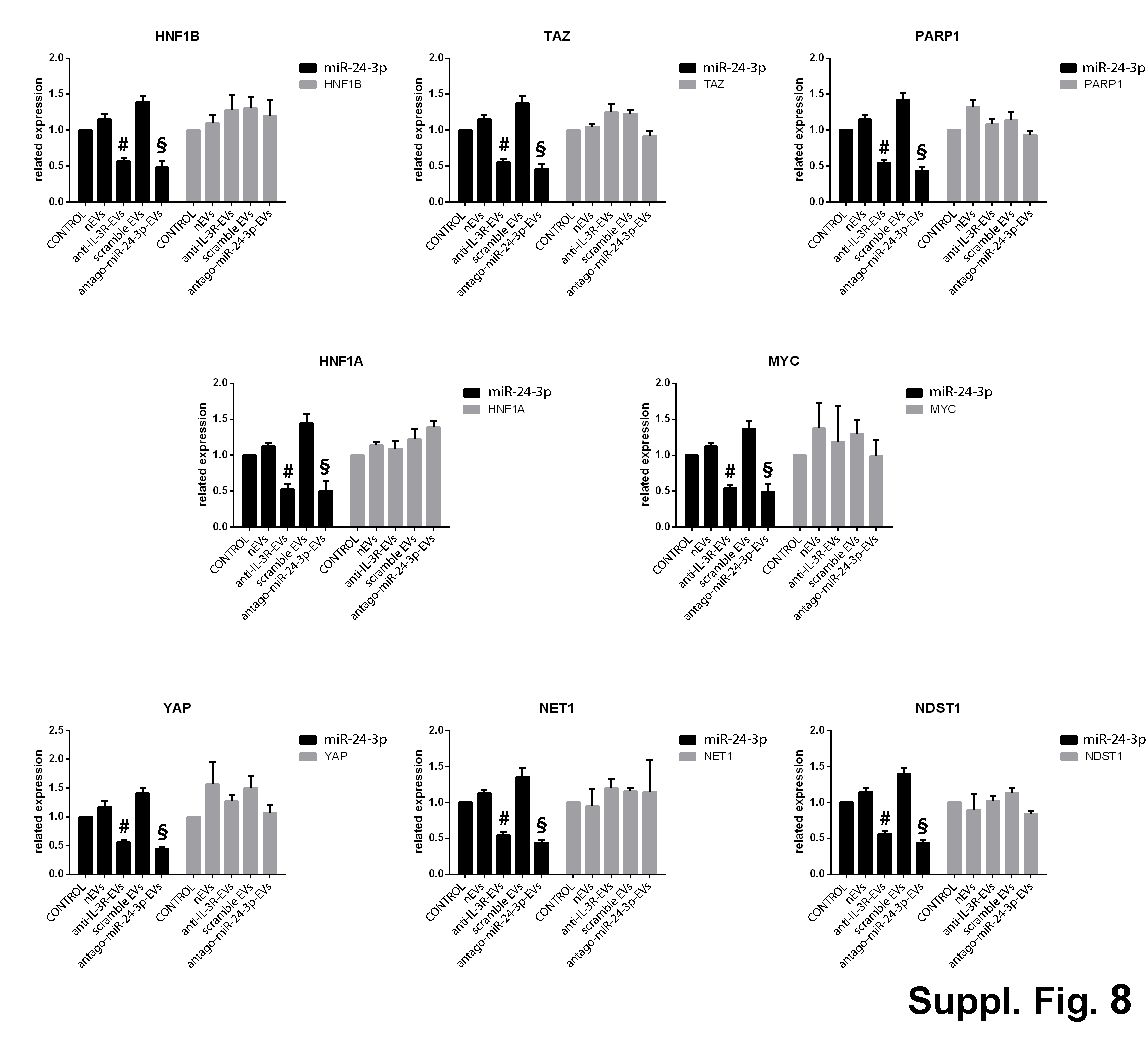
**

**Supplementary Fig. S8. miR-24-3p target gene validation.** qRT-PCR for HNF1B, TAZ, PARP1, HNF1A, MYC, YAP, NET1, NDST1 on MDA-MB-231 cells untreated (CONTROL) and treated with nEVs, anti-IL-3R-EVs, scramble EVs and antago-miR-24-3p-EVs. Related expression of miR-24-3p for each condition is reported in each graph (n=3). # p< 0.05 anti-IL-3R-EVs vs nEVs; §p< 0.05 antago-miR-24-3p-EVs vs scramble EVs.

**Supplementary Fig. S9**
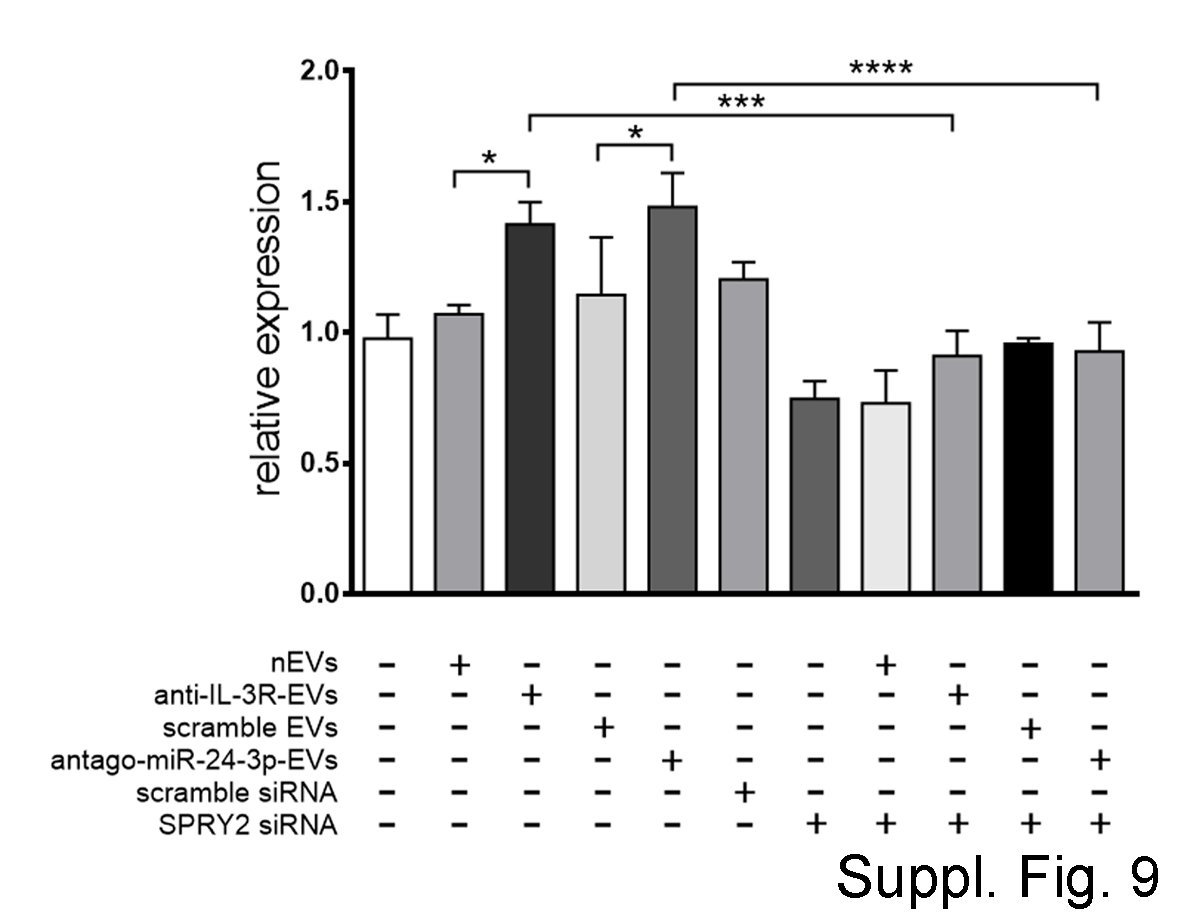


**Supplementary Fig. S9. SPRY2 expression in MDA-MB-231.** qRT-PCR for SPRY2 in MDA-MB-231 cells untransfected or transfected with SPRY2 siRNA unstimulated or subjected to nEVs, anti-IL-3R-EVs, scramble EVs or antago-miRNA-24-3p-EVs. Data are expressed as relative expression as indicated ±SD (n=6). *p<0.05: nEVs vs anti-IL-3R-EVs, scramble EVs vs antago-miRNA-24-3p-EVs; ***p<0.005: anti-IL-3R-EVs vs SPRY2 silenced cells + anti-IL-3R-EVs; ****p<0.0001: antago-miRNA-24-3p-EVs vs SPRY2 silenced cells + antago-miRNA-24-3p-EVs.

**Supplementary Table S1. Features of human TNBC samples (n=27).**

| **Characteristic/Parameter** | **Type** | **Number of cases** |
| --- | --- | --- |
| Histotype | Ductal  Lobular  others | 20  2  5 |
| Histological grade | 2  3 | 4  23 |
| Size (mm) | 10-20  20-50  >50 | 7  3  17 |
| Vascular invasion | No  Yes | 14  13 |
| Lymph node involvement | No  Yes | 12  15 |

**Supplementary Table S2. List of primers.**

| **Gene Name** | **Forward** | **Reverse** |
| --- | --- | --- |
| **hTWIST1** | F1: GTCCGCAGTCTTACGAGGAG | R1: GCTTGAGGGTCTGAATCTTGCT |
| **hHNF1A** | F1: CTTCTGCAGGAGGACCCGT | R1: CCCGCTTCTGCGTCTTC |
| **hPARP1** | F1: GATGGCCATCATGGTGCAG | R1: CGCTGTCTTCTTGACTTTCTGCT |
| **hNDST1** | F1: TCTTCAAGGCCAATGAGAACAG | R1: TTGACTGGAAAACCGTCCAGTC |
| **hNET1** | F1: AGCTGGTTACCGCGCTTG | R1: ACTAGGCGACTTCGAGGGATATCTA |
| **hTAZ** | F1: CTGGACCAAGTACATGAACCACC | R1: CGCATCAACTTCAGGTTCCAG |
| **hYAP** | F1: CCCGACAGGCCAGTACTGAT | R1: AAAGAAGACTGTGAAGATGCTGAG |
| **hMYC** | F1: TGGAAACCCGATGAAATACA | R1: GTCGCAGATGAAATAGGGCTG |
| **hSPRY2** | F1: GCGATCACGGAGTTCAGATGT | R1: GGCTCCCCACGCTGTCT |
| **hHNF1B** | F1: TCACAGATACCAGCAGCATCA | R1: GGGCATCACCAGGCTTGTA |

**Detailed Methods**

**Immunohistochemistry and immunofluorescence on human and animal samples**

A series of 27 patients diagnosed with triple negative breast cancer (TNBC) between 2011 and 2012 was retrieved from the files of the Pathology Department of the Città della Salute e della Scienza Hospital (Turin). All cases were anonymously recorded into a dedicated database. The study was conducted in accordance with The Code of Ethics of the World Medical Association (Declaration of Helsinki) and within the guidelines and regulations defined by the Research Ethics Committee for human Biospecimen Utilization (Department of Medical Sciences—ChBU) of the University of Turin. The human study was retrospective, it included already existing medical data which were previously anonymized, and have no impact on patient’s care. Our local Ethical Committee did not require specific written informed consent. Pathological data, including histotype, grade and size of the tumors, and peritumoral vascular invasion, were recorded in accordance with the original diagnosis (Supplementary Table S1). Representative blocks were obtained and multicore tissue microarrays (TMAs) prepared, using the advanced tissue array (mod ATA-100, Chemicon International, Tamecula, CA, USA). Immunohistochemistry was performed using an automated slide-processing platform (Ventana BenchMark AutoStainer, Ventana Medical Systems, Tucson, AZ, USA), with Universal DAB Detection Kit detection systems. The anti-IL-3Rα clone S-12 antibody (sc-455 Santa Cruz Biotechnology, Dallas, TX) was used at a dilution of 1:50. After microwave heating for 36 min, sections were incubated for 30 min with the primary antibody. The primary acute myeloid-derived cell line, MO7, served as positive controls and were included for each immunohistochemical run.

5 µm paraffin-embedded tumor sections were stained with anti-CD31 antibody (AF3628 R&D System) followed by PAS staining (BioOptica) to quantify CD31+/PAS+ vessels and evaluate the vasculogenic mimicry. Ten sections/tumors were analyzed using ImageJ and vessel CD31+/PAS+ and vascular mimicry CD31-/PAS+ were quantified as the number of CD31+PAS+ /field and CD31-PAS+/field respectively. In addition, paraffin-embedded tumor sections were stained with Masson’s trichrome to quantify all the vessels with red blood cells inside. Ten sections/tumors were analyzed using ImageJ software and the results were expressed as vessel/field ±SD in arbitrary units. Moreover, tumor sections were analyzed using the ApopTag®Plus Peroxidase In Situ Apoptotic Detection kit (Millipore, #S7101), following the manufacture’s protocol, to evaluate apoptotic tumor cells. The number of TUNEL-positive nuclei was evaluated by counting the number of positive nuclei per field in 10 randomly chosen sections of tumor (Original magnification 20X) using ImageJ software. Immunohistochemistry for the detection of Vimentin, TWIST1 and β-catenin was performed using a monoclonal anti-Vimentin antibody (Sigma #V5255), a polyclonal anti-TWIST1 antibody (Abcam #ab49254) and a polyclonal anti-β-catenin antibody (Abcam #ab16051). Immunoperoxidase reactions were performed using appropriate HRP-conjugated secondary antibodies (Pierce, Rockford, IL). Quantifications of Vimentin and TWIST1 positive area were performed using Fiji software; the computer automatically detected the positive area with the same color configuration as the photo and converted the data to a percentage of the total area in each field. Ten randomly selected tumor sections were analyzed and the results were expressed as percentage of positive area/total area ±SD. The analysis of β-catenin positive cells was performed by 2 independent pathologists and the results were classified into 3 categories according to β-catenin subcellular localization: nuclear, cytoplasmic or membranous. All slides were given a score representing the estimated proportion (0: <5%, 1: 5%–25%, 2: 25%–75%, and 3: >75%) of positively stained (P) cancer cells, while the intensity (I) scores 1, 2, or 3 were assigned to weak, moderate and strong staining, respectively. The Quick score (Q) was calculated by multiplying the percentage of positive cells (P) by the intensity (I), according to the formula: Q = P x I [43]. The livers and lungs were analyzed for the presence of metastasis. MDA-MB-231 cells were detected by immunofluorescence using anti-HLA I (Santa Cruz Biotechnology, #sc-25619). Four randomly chosen tumor sections were analyzed by counting 10 fields/section/group (n=4 samples/each group). Primary tumors and mouse livers were used as positive and negative controls, respectively. The counting of HLA I+ cells was performed using ImageJ software and results were expressed as percentage of HLA I+ cells with respect to total cells/field ±SD (lung) and as percentage of HLA I+ cells/total area (liver).

## **Cell cultures**

The MDA-MB-231 and MDA-MB-453 and MCF10A cell lines were purchased from ATCC and cultured in the DMEM medium in the presence of 10% FBS and 100 U/ml of penicillin/streptomycin. The incubation was performed at 37°C with 5% CO_2_ and controlled humidity. Human derived TEC were obtained in our laboratory from surgical tumor specimens using anti-CD105 positive selection with magnetic cell sorting (MACS system, Miltenyi Biotech) and were cultured in EndoGro complete medium (Millipore). Primary human umbilical vein endothelial cells (HUVEC), purchased from ATCC and used as controls of non-tumoral endothelial cells, were either untreated or treated with IL-3 (10 ng/ml) to obtain EVs (EV ctr and EV IL-3 respectively).

## **EV isolation and characterization**

In selected experiments, starved TEC were cultured (24h) in the presence of 1 μg/ml Human IL-3Rα/CD123 MAb (R&D Systems, #MAB301-100, Clone 32703). Untreated TEC served as controls. For EV isolation, TEC, untreated or pre-treated by blocking IL-3Rα, were cultured for 24h in FBS-free EndoGro medium. The conditioned medium was centrifuged for 30 min at 3,000g to remove cell debris and apoptotic bodies, and then submitted to microfiltration with 0.22 μm filters (MF-Millipore™) to remove larger vesicles. The supernatant was then ultracentrifuged for 2h at 100,000g, 4°C, using the Beckman Coulter Optima L-100K Ultracentrifuge with the rotor type 45 Ti 45,000 rpm. The pellet of EVs obtained was resuspended in DMEM supplemented with 1% DMSO. The TEC-EV suspension was then stored at -80°C until further use.

In specific experiments, TEC were transfected with antago-miR-24-3p (Ambion, cat #4464085, assay ID MH1073) or scramble SiRNA (Ambion, cat #4464077). Transfection was performed with the Lipofectamine® RNAiMAX Reagent (Thermofisher), according to the manufacturer’s instructions. TEC were seeded in T175 flasks in EndoGro complete medium. For the transfection, we mixed a suspension of 62.5 μl of Lipofectamine® RNAiMAX Reagent in 3 ml of Optimem (Gibco) with 1.25 μM of either the antago-miR-24-3p or scramble miRNA in 3 ml of Optimem. After 5 min of incubation, the transfection mix was added to cells in complete medium. After 24h, the medium was changed to the FBS-free EndoGro medium for an additional 24h, which is the time required for EV production.

**EV characterization**

EVs were analyzed using NTA, the NanoSight NS300 system (Malvern Instruments, Ltd), electron microscopy and FACS analysis. Transmission electron microscopy (TEM) was performed on EVs placed on 200-mesh nickel formvar carbon-coated grids (Electron Microscopy Science) for 20 min to promote adhesion. The grids were then incubated with 2.5% glutaraldehyde plus 2% sucrose, EVs were negatively stained with NanoVan (Nanoprobes, Yaphank, NY, USA) after washings, and observed using a Jeol JEM 1010 electron microscope (Jeol, Tokyo, Japan). Moreover, EV flow cytometry analysis was performed using the MACSPlex Exosome Kit (human, Miltenyi Biotec), following the manufacturer’s protocol. Briefly, 10 µl of each sample was diluted with MACSPlex buffer to a final volume of 120 μl and 15 μl of MACSPlex Exosome Capture Beads (containing different antibody-coated bead subsets) was added to each sample. Next, samples were incubated overnight, protected from light, on a rotator and an orbital shaker at 450 rpm at room temperature. To wash the beads, 1 ml of MACSPlex buffer was added to each tube and centrifuged at 3,000g for 5 min. After the supernatant was removed, EVs were bound by capture beads with detection antibodies and 5 μl of each APC-conjugated anti-CD9, anti-CD81 and anti-CD63 detection antibody were incubated on an orbital shaker at 450 rpm, protected from light, for 1h at room temperature. Samples were washed with 1 ml of MACSPlex buffer and centrifuged at 3,000g for 5 min. After the supernatant was removed, each tube was incubated for 15 min with 1 ml of MACSPlex buffer on an orbital shaker at 450 rpm, protected from light, for 1h at room temperature. After being centrifuged at 3,000g for 5 min, the supernatant was aspirated and the remaining 150 µl of each sample was analyzed using a CytoFLEX® Flow Cytometer (Beckman Coulter). CytExpert Software (Beckman Coulter) was used to analyze flow cytometric data, and the median APC fluorescence intensity (MFI) for all 39 capture bead subsets was background corrected by subtracting the respective MFI values from the matched media control (medium + capture beads + antibodies). Statistical analysis was performed using two-way ANOVA with Sidak's multiple comparisons test. EVs were also analyzed for the CD63 exosomal marker by western blot.

## **Cell counting, apoptosis, scratch test, SPRY2 silensing**

For the apoptosis assay, cells were seeded in 6-well plates and then stimulated with different types of TEC-EVs (nEVs, anti-IL-3R-EVs, scramble EVs, antago-miR-24-3p-EVs) at the concentration 2×10^8^ EVs/ml in FBS-free DMEM for 24h. The effective dose was selected with reference to the preliminary results obtained using different EV concentrations (data not shown). Treated and untreated cells were analyzed using Muse® Annexin V & Dead Cell Kit (Millipore, #MCH100105). Cell proliferation was assayed by direct cell count by two different operators. In particular, cells were unstimulated or stimulated with different TEC-EVs (2×10^8^ EVs/ml) cultured for 48h in DMEM FBS-free medium and counted. In selected experiments, direct cell count was also applied to evaluate MCF10A cell proliferation. Cells were unstimulated or stimulated with different TEC-EVs (2×10^8^ EVs/ml) cultured for 48h in DMEM FBS-free medium and counted. For the scratch assay, cells were seeded in 24-well plates and grown until confluence in DMEM 10% FBS. Cells were then stimulated with TEC-EVs (nEVs, anti-IL-3R-EVs, scramble EVs, antago-miR-24-3pEVs) in DMEM FBS-free medium. The scratch assay was performed 24h later using a 200 μl micropipette tip. Images were captured using a light microscope at 0 and 24h after the scratch, and the distance was measured using LAS software (Leica). Results were expressed as mean distance (0-24h) ±SD of six independent experiments. In selected experiments SPRY2 was silenced in MDA-MB-231 cells by transfecting siRNA scramble (Qiagen, Cat No 1027310) or siRNA for SPRY2 (Qiagen, Cat No SI00081788) using HiPerFect Transfection Reagent (Qiagen, Cat No 301704), following manufacturer’s instructions. Briefly, cells were seeded 6×10^4^ cells in 24 well plate and transfected with 10nM of siRNA using 3 µl of HiPerFect in a final volume of 600 µl/well. 30 hours later the cells were stimulated with nEVs, anti-IL-3R-EVs, scramble EVs, antago-miR-24-3pEVs and analyzed for apoptosis as above reported. Real-Time PCR was used to verify SPRAY2 silencing by and miR-24-3p using the indicated primers.

**Sphere formation assay**

To test the ability of MDA-MB-231 cells to grow in non-adhesive conditions as floating spheres, cells were detached by a non-enzymatic solution (Merck), washed twice with DMEM FBS-free, counted and plated in 6-well non-adherent plates, at a concentration 50×10^3^/well, in 2 ml of sphere formation medium (Dulbecco’s Modified Eagle Medium/F12 completed with 20 ng/ml EGF, 20 ng/ml EGF, 5 μg/ml insulin and 0.4% Bovine Serum Albumin) in the presence of nEVs or anti-IL-3R-EVs (1×10^8^ EVs/ml) for 72h without disturbing. At day 3 and 6, 0.5 ml of culture medium containing nEVs and anti-IL-3R-EVs was added. On day 10, the number of spheres for each well were counted using light microscopy. Data are expressed as number of sphere/sample ±SD of three different experiments performed in duplicate.

## **Tumor growth and model of metastasis formation *in vivo***

Animal studies were conducted in accordance with the Italian National Institute of Health Guide for the Care and Use of Laboratory Animals (protocol no: 944/2015-PR). Mice were housed according to the guidelines of the Federation of European Laboratory Animal Science Association and the Ethical Committee of the University of Turin. The experimental studies did not include client-owned animals. The investigators (at least 2) were blinded when assessing the outcome.

Tumors were obtained by injecting MDA-MB-231 cells in Matrigel into the mammary fat pad of SCID mice (4 mice/group) (1×10^6^ cells per injection). SCID female mice (Charles River Laboratories), aged 8 weeks were used. After 3 weeks, when tumors became palpable, animals were treated with nEVs or anti-IL-3R-EVs (1×10^10^ EV per tumor) twice a week for 3 additional weeks (Fig. 3A), as control saline was used. At day 45, tumors were excised, fixed in 10% buffered neutral formalin and embedded in paraffin (n= 4/each condition). To evaluate the effects of nEVs, either anti-IL-3R-EVs, or antago-miR-24-3p-EVs in metastasis formation after intravenous tumor injection, EVs (1×10^10^ EV/injection) were intravenously injected for 5 days into SCID mice (Fig. 5A). On day 5, 0.6×10^6^ MDA-MB-231 cells were injected intravenously. The mice were sacrificed after 5 weeks; the lungs were fixed in 10% buffered neutral formalin and embedded in paraffin. Five µm sections were analyzed by hematoxylin and eosin staining. Lung metastases were counted using ImageJ in 5 non-sequential sections. Results were expressed as mean ±SD of metastasis per lung (n=4/each condition). In addition, lung vessels with red blood cells inside were quantified in paraffin-embedded lung sections stained with Masson’s trichrome and expressed as vessels/field ±SD.

## **Real-time PCR**

Real-time PCR was performed to detect miR-24-3p (in transfected TEC-EVs) while TWIST1 and SPRY2 in MDA-MB-231 cells after treatment. Total RNA from TEC-EV samples and MDA-MB-231 cells was extracted using the RNAeasy kit (Qiagen). RNA was reverse transcribed into complementary DNA (cDNA) using miScript II RT Kit (Qiagen), according to the manufacturer’s protocol using hiflex buffer applicable for mRNA and miR PCR detection. Real-time PCR was performed using the miScript SYBR Green PCR kit (Qiagen). Specific TWIST1 primers were obtained from the primer bank (<https://pga.mgh.harvard.edu/primerbank/>) (Supplementary Table S2). All samples were run in triplicate using 3 ng of cDNA for each reaction, as described in the manufacturer's protocol (Qiagen). Relative expression data were normalized using the expression value of two housekeeping genes (actin and 18S RNA), according to a Ct detection cut-off of 35 PCR cycles.

## **Western Blot**

Protein samples of tumor cells treated with the various TEC-EVs types were separated by 4% to 20% gradient sodium dodecyl sulfate–polyacrylamide gel electrophoresis (SDS PAGE, Biorad) and subjected to immunoblotting using an anti-TWIST1 antibody (Abcam, #ab49254) and an anti-Actin antibody (Santa Cruz Biotechnology, #sc-47778) (n=3). The protein was visualized using an enhanced chemiluminescence detection kit and ChemiDoc™ XRS+ System (BioRad). MDA-MB-231 cells were also evaluated for their IL-3Rα expression using the IL-3Rα/CD123 MAb (R&D Systems, #MAB301-100, Clone 32703). Cell lysates (30 μg protein) were loaded for western blot analysis. In selected experiment lysed TEC EVs (nEVs and anti-IL-3R-EVs) (50 μg protein) were also analyzed for the expression of the CD63 exosomal marker using the mouse monoclonal antibody (sc-5275 Santa Cruz Biotechnology). Anti-E-cadherin (Santa Cruz Biotechnology, #sc 7870) and anti-N- cadherin (Abcam, #ab18203) antibody were used in selected experiments (n=3). In selected experiments, anti-GAPDH and anti-Vinculin antibody was used as housekeeping (sc-47724 Santa Cruz Biotechnology, Millipor MAB3574 respectively).

## **miR-24-3p target validation**

Ingenuity pathway analysis (IPA) generates networks based on the Ingenuity Knowledge Base, which relies on known biological gene interplay. IPA was used to predict the target genes for miR-24-3p. The miR Target Filter tool was set up on IPA (Qiagen: [**http://www.qiagenbioinformatics.com/products/ingenuity-pathway-analysis/**](http://www.qiagenbioinformatics.com/products/ingenuity-pathway-analysis/)) to associate miR-24-3p with predicted mRNA targets. miR-24-3p target expression was evaluated by RT-PCR. Total RNA from MDA-MB-231 that was treated with nEVs, anti-IL-3R-EVs and either transfected with scrambled random sequence (scrambled EVs) or with antago-miR-24-3p-EVs was extracted using All in One (Norgen, Thorold, ON, Canada) and analyzed with a NanoDrop1000 spectrophotometer; samples of absorbance at 260/280 nm between 1.8 and 2.0 were adopted. We evaluated HNF1B, TAZ, PARP1, HNF1A, SPRY2, MYC, YAP, NET1 and NDST1 mRNA expression using the High cDNA Reverse Transcription Kit and Power SYBR Green PCR Master Mix on the StepOnePlus Real Time System (Applied Biosystems, USA) using β-actin as the housekeeping transcript. Primer sequences are reported in Supplementary Table S2. Changes in RNA expression were calculated using the 2^-ΔΔCt^ method. Statistical analysis was performed with One-way ANOVA, followed by Tukey’s multiple comparison test.
