# Supplementary material for: Targeting IL-3Rα on tumor-derived endothelial cells blunts metastatic spread of triple-negative breast cancer via extracellular vesicle reprogramming
Source: Oncogenesis. 2020 Oct 10;9(10):90. doi: 10.1038/s41389-020-00274-y (PMC7548009; doi:10.1038/s41389-020-00274-y)
Supplement: Supplementary file 2 — authorship form [file 41389_2020_274_MOESM2_ESM.pdf]

SPRINGER NATURE

In accordance with Springer Nature Authorship Policy we agree to change the authors of the manuscript as indicated below.

NAME OF JOURNAL: Oncogenesis

TITLE OF MANUSCRIPT: Targeting IL-3Rα on tumor-derived endothelial cells blunts metastatic spread of triple negative breast cancer via extracellular vesicle reprogramming

MANUSCRIPT NUMBER: ONCSIS-20-0332R

CORRESPONDING AUTHORS NAME: Maria Felice Brizzi

PREVIOUS AUTHOR NAMES:

Tatiana Lopatina1, Cristina Grange1, Claudia Cavallari2, Victor Navarro-Tableros2, Giusy Lombardo1, Arturo Rosso1, Massimo Cedrino2, Margherita Alba Carlotta Pomatto1, Francesca Veneziano1, Isabella Castellano1, Giovanni Camussi1*, and Maria Felice Brizzi1*

UPDATED AUTHOR NAMES:

Malvina Koni has been included in the present version of the MS since she has performed biochemical and siRNA experiments requested by Reviewers

CHANGE TO AUTHOR LIST:

Tatiana Lopatina1, Cristina Grange1, Claudia Cavallari2, Victor Navarro-Tableros2, Giusy Lombardo1, Arturo Rosso1, Massimo Cedrino2, Margherita Alba Carlotta Pomatto1, Malvina Koni1, Francesca Veneziano1, Isabella Castellano1, Giovanni Camussi1*, and Maria

Print Name	Signature	Date
Tatiana Lopatina	TLopatina	08/06/2020

SPRINGER NATURE

In accordance with Springer Nature Authorship Policy we agree to change the authors of the manuscript as indicated below.

NAME OF JOURNAL: Oncogenesis

TITLE OF MANUSCRIPT: Targeting IL-3Rα on tumor-derived endothelial cells blunts metastatic spread of triple negative breast cancer via extracellular vesicle reprogramming

MANUSCRIPT NUMBER: ONCSIS-20-0332R

CORRESPONDING AUTHORS NAME: Maria Felice Brizzi

PREVIOUS AUTHOR NAMES:

Tatiana Lopatina1, Cristina Grange1, Claudia Cavallari2, Victor Navarro-Tableros2, Giusy Lombardo1, Arturo Rosso1, Massimo Cedrino2, Margherita Alba Carlotta Pomatto1, Francesca Veneziano1, Isabella Castellano1, Giovanni Camussi1*, and Maria Felice Brizzi1*

UPDATED AUTHOR NAMES:

Malvina Koni has been included in the present version of the MS since she has performed biochemical and siRNA experiments requested by Reviewers

CHANGE TO AUTHOR LIST:

Tatiana Lopatina1, Cristina Grange1, Claudia Cavallari2, Victor Navarro-Tableros2, Giusy Lombardo1, Arturo Rosso1, Massimo Cedrino2, Margherita Alba Carlotta Pomatto1, Malvina Koni1, Francesca Veneziano1, Isabella Castellano1, Giovanni Camussi1*, and Maria

Print Name	Signature	Date
Cristina Grange	Cristina Grange	08/06/2020

SPRINGER NATURE

In accordance with Springer Nature Authorship Policy we agree to change the authors of the manuscript as indicated below.

NAME OF JOURNAL: Oncogenesis

TITLE OF MANUSCRIPT: Targeting IL-3Rα on tumor-derived endothelial cells blunts metastatic spread of triple negative breast cancer via extracellular vesicle reprogramming

MANUSCRIPT NUMBER: ONCSIS-20-0332R

CORRESPONDING AUTHORS NAME: Maria Felice Brizzi

PREVIOUS AUTHOR NAMES:

Tatiana Lopatina1, Cristina Grange1, Claudia Cavallari2, Victor Navarro-Tableros2, Giusy Lombardo1, Arturo Rosso1, Massimo Cedrino2, Margherita Alba Carlotta Pomatto1, Francesca Veneziano1, Isabella Castellano1, Giovanni Camussi1*, and Maria Felice Brizzi1*

UPDATED AUTHOR NAMES:

Malvina Koni has been included in the present version of the MS since she has performed biochemical and siRNA experiments requested by Reviewers

CHANGE TO AUTHOR LIST:

Tatiana Lopatina1, Cristina Grange1, Claudia Cavallari2, Victor Navarro-Tableros2, Giusy Lombardo1, Arturo Rosso1, Massimo Cedrino2, Margherita Alba Carlotta Pomatto1, Malvina Koni1, Francesca Veneziano1, Isabella Castellano1, Giovanni Camussi1*, and Maria

Print Name	Signature	Date
Claudia Cavallari	Claudia Cavallari	08/06/2020

SPRINGER NATURE

In accordance with Springer Nature Authorship Policy we agree to change the authors of the manuscript as indicated below.

NAME OF JOURNAL: Oncogenesis

TITLE OF MANUSCRIPT: Targeting IL-3Rα on tumor-derived endothelial cells blunts metastatic spread of triple negative breast cancer via extracellular vesicle reprogramming

MANUSCRIPT NUMBER: ONCSIS-20-0332R

CORRESPONDING AUTHORS NAME: Maria Felice Brizzi

PREVIOUS AUTHOR NAMES:

Tatiana Lopatina1, Cristina Grange1, Claudia Cavallari2, Victor Navarro-Tableros2, Giusy Lombardo1, Arturo Rosso1, Massimo Cedrino2, Margherita Alba Carlotta Pomatto1, Francesca Veneziano1, Isabella Castellano1, Giovanni Camussi1*, and Maria Felice Brizzi1*

UPDATED AUTHOR NAMES:

Malvina Koni has been included in the present version of the MS since she has performed biochemical and siRNA experiments requested by Reviewers

CHANGE TO AUTHOR LIST:

Tatiana Lopatina1, Cristina Grange1, Claudia Cavallari2, Victor Navarro-Tableros2, Giusy Lombardo1, Arturo Rosso1, Massimo Cedrino2, Margherita Alba Carlotta Pomatto1, Malvina Koni1, Francesca Veneziano1, Isabella Castellano1, Giovanni Camussi1*, and Maria

Print Name	Signature	Date
Victor Navarro-Tableros	[illegible]	08/06/2020

SPRINGER NATURE

In accordance with Springer Nature Authorship Policy we agree to change the authors of the manuscript as indicated below.

NAME OF JOURNAL: Oncogenesis

TITLE OF MANUSCRIPT: Targeting IL-3Rα on tumor-derived endothelial cells blunts metastatic spread of triple negative breast cancer via extracellular vesicle reprogramming

MANUSCRIPT NUMBER: ONCSIS-20-0332R

CORRESPONDING AUTHORS NAME: Maria Felice Brizzi

PREVIOUS AUTHOR NAMES:

Tatiana Lopatina1, Cristina Grange1, Claudia Cavallari2, Victor Navarro-Tableros2, Giusy Lombardo1, Arturo Rosso1, Massimo Cedrino2, Margherita Alba Carlotta Pomatto1, Francesca Veneziano1, Isabella Castellano1, Giovanni Camussi1*, and Maria Felice Brizzi1*

UPDATED AUTHOR NAMES:

Malvina Koni has been included in the present version of the MS since she has performed biochemical and siRNA experiments requested by Reviewers

CHANGE TO AUTHOR LIST:

Tatiana Lopatina1, Cristina Grange1, Claudia Cavallari2, Victor Navarro-Tableros2, Giusy Lombardo1, Arturo Rosso1, Massimo Cedrino2, Margherita Alba Carlotta Pomatto1, Malvina Koni1, Francesca Veneziano1, Isabella Castellano1, Giovanni Camussi1*, and Maria

Print Name	Signature	Date
Giusy Lombardo	Giusy Lombardo	08/06/2020

SPRINGER NATURE

In accordance with Springer Nature Authorship Policy we agree to change the authors of the manuscript as indicated below.

NAME OF JOURNAL: Oncogenesis

TITLE OF MANUSCRIPT: Targeting IL-3Rα on tumor-derived endothelial cells blunts metastatic spread of triple negative breast cancer via extracellular vesicle reprogramming

MANUSCRIPT NUMBER: ONCSIS-20-0332R

CORRESPONDING AUTHORS NAME: Maria Felice Brizzi

PREVIOUS AUTHOR NAMES:

Tatiana Lopatina1, Cristina Grange1, Claudia Cavallari2, Victor Navarro-Tableros2, Giusy Lombardo1, Arturo Rosso1, Massimo Cedrino2, Margherita Alba Carlotta Pomatto1, Francesca Veneziano1, Isabella Castellano1, Giovanni Camussi1*, and Maria Felice Brizzi1*

UPDATED AUTHOR NAMES:

Malvina Koni has been included in the present version of the MS since she has performed biochemical and siRNA experiments requested by Reviewers

CHANGE TO AUTHOR LIST:

Tatiana Lopatina1, Cristina Grange1, Claudia Cavallari2, Victor Navarro-Tableros2, Giusy Lombardo1, Arturo Rosso1, Massimo Cedrino2, Margherita Alba Carlotta Pomatto1, Malvina Koni1, Francesca Veneziano1, Isabella Castellano1, Giovanni Camussi1*, and Maria

Print Name	Signature	Date
Arturo Rosso		08/06/2020

SPRINGER NATURE

In accordance with Springer Nature Authorship Policy we agree to change the authors of the manuscript as indicated below.

NAME OF JOURNAL: Oncogenesis

TITLE OF MANUSCRIPT: Targeting IL-3Rα on tumor-derived endothelial cells blunts metastatic spread of triple negative breast cancer via extracellular vesicle reprogramming

MANUSCRIPT NUMBER: ONCSIS-20-0332R

CORRESPONDING AUTHORS NAME: Maria Felice Brizzi

PREVIOUS AUTHOR NAMES:

Tatiana Lopatina1, Cristina Grange1, Claudia Cavallari2, Victor Navarro-Tableros2, Giusy Lombardo1, Arturo Rosso1, Massimo Cedrino2, Margherita Alba Carlotta Pomatto1, Francesca Veneziano1, Isabella Castellano1, Giovanni Camussi1*, and Maria Felice Brizzi1*

UPDATED AUTHOR NAMES:

Malvina Koni has been included in the present version of the MS since she has performed biochemical and siRNA experiments requested by Reviewers

CHANGE TO AUTHOR LIST:

Tatiana Lopatina1, Cristina Grange1, Claudia Cavallari2, Victor Navarro-Tableros2, Giusy Lombardo1, Arturo Rosso1, Massimo Cedrino2, Margherita Alba Carlotta Pomatto1, Malvina Koni1, Francesca Veneziano1, Isabella Castellano1, Giovanni Camussi1*, and Maria

Print Name	Signature	Date
Massimo Cedrino	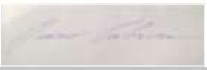	08/06/2020

SPRINGER NATURE

In accordance with Springer Nature Authorship Policy we agree to change the authors of the manuscript as indicated below.

NAME OF JOURNAL: Oncogenesis

TITLE OF MANUSCRIPT: Targeting IL-3Rα on tumor-derived endothelial cells blunts metastatic spread of triple negative breast cancer via extracellular vesicle reprogramming

MANUSCRIPT NUMBER: ONCSIS-20-0332R

CORRESPONDING AUTHORS NAME: Maria Felice Brizzi

PREVIOUS AUTHOR NAMES:

Tatiana Lopatina1, Cristina Grange1, Claudia Cavallari2, Victor Navarro-Tableros2, Giusy Lombardo1, Arturo Rosso1, Massimo Cedrino2, Margherita Alba Carlotta Pomatto1, Francesca Veneziano1, Isabella Castellano1, Giovanni Camussi1*, and Maria Felice Brizzi1*

UPDATED AUTHOR NAMES:

Malvina Koni has been included in the present version of the MS since she has performed biochemical and siRNA experiments requested by Reviewers

CHANGE TO AUTHOR LIST:

[illegible], [illegible], Claudia Cavallari2, Victor Navarro-Tableros2, Giusy Lombardo1, Arturo Rosso1, Massimo Cedrino2, Margherita Alba Carlotta Pomatto1, Malvina Koni1, Francesca Veneziano1, Isabella Castellano1, Giovanni Camussi1*, and Maria

Print Name	Signature	Date
Margherita Alba Carlotta Pomatto	Margherita Alba Carlotta Pomatto	08/06/2020

SPRINGER NATURE

In accordance with Springer Nature Authorship Policy we agree to change the authors of the manuscript as indicated below.

NAME OF JOURNAL: Oncogenesis

TITLE OF MANUSCRIPT: Targeting IL-3Rα on tumor-derived endothelial cells blunts metastatic spread of triple negative breast cancer via extracellular vesicle reprogramming

MANUSCRIPT NUMBER: ONCSIS-20-0332R

CORRESPONDING AUTHORS NAME: Maria Felice Brizzi

PREVIOUS AUTHOR NAMES:

Tatiana Lopatina1, Cristina Grange1, Claudia Cavallari2, Victor Navarro-Tableros2, Giusy Lombardo1, Arturo Rosso1, Massimo Cedrino2, Margherita Alba Carlotta Pomatto1, Francesca Veneziano1, Isabella Castellano1, Giovanni Camussi1*, and Maria Felice Brizzi1*

UPDATED AUTHOR NAMES:

Malvina Koni has been included in the present version of the MS since she has performed biochemical and siRNA experiments requested by Reviewers

CHANGE TO AUTHOR LIST:

Tatiana Lopatina1, Cristina Grange1, Claudia Cavallari2, Victor Navarro-Tableros2, Giusy Lombardo1, Arturo Rosso1, Massimo Cedrino2, Margherita Alba Carlotta Pomatto1, Malvina Koni1, Francesca Veneziano1, Isabella Castellano1, Giovanni Camussi1*, and Maria

Print Name	Signature	Date
Malvina Koni	Malvina Koni	08/06/2020

SPRINGER NATURE

In accordance with Springer Nature Authorship Policy we agree to change the authors of the manuscript as indicated below.

NAME OF JOURNAL: Oncogenesis

TITLE OF MANUSCRIPT: [illegible]

MANUSCRIPT NUMBER: ONCSIS-20-0332R

CORRESPONDING AUTHORS NAME: Maria Felice Brizzi

PREVIOUS AUTHOR NAMES:

Tatiana Lopatina1, Cristina Grange1, Claudia Cavallari2, Victor Navarro-Tableros2, Giusy Lombardo1, Arturo Rosso1, Massimo Cedrino2, Margherita Alba Carlotta Pomatto1, Francesca Veneziano1, Isabella Castellano1, Giovanni Camussi1*, and Maria Felice Brizzi1*

UPDATED AUTHOR NAMES:

Malvina Koni has been included in the present version of the MS since she has performed biochemical and siRNA experiments requested by Reviewers

CHANGE TO AUTHOR LIST:

[illegible]

Print Name	Signature	Date
Francesca Veneziano	[illegible]	08/06/2020

SPRINGER NATURE

In accordance with Springer Nature Authorship Policy we agree to change the authors of the manuscript as indicated below.

NAME OF JOURNAL: Oncogenesis

TITLE OF MANUSCRIPT: [illegible]

MANUSCRIPT NUMBER: ONCSIS-20-0332R

CORRESPONDING AUTHORS NAME: Maria Felice Brizzi

PREVIOUS AUTHOR NAMES:

Tatiana Lopatina1, Cristina Grange1, Claudia Cavallari2, Victor Navarro-Tableros2, Giusy Lombardo1, Arturo Rosso1, Massimo Cedrino2, Margherita Alba Carlotta Pomatto1, Francesca Veneziano1, Isabella Castellano1, Giovanni Camussi1*, and Maria Felice Brizzi1*

UPDATED AUTHOR NAMES:

Malvina Koni has been included in the present version of the MS since she has performed biochemical and siRNA experiments requested by Reviewers

CHANGE TO AUTHOR LIST:

[illegible]

Print Name	Signature	Date
Isabella Castellano	[illegible]	08/06/2020

SPRINGER NATURE

In accordance with Springer Nature Authorship Policy we agree to change the authors of the manuscript as indicated below.

NAME OF JOURNAL: Oncogenesis

TITLE OF MANUSCRIPT: Targeting IL-3Rα on tumor-derived endothelial cells blunts metastatic spread of triple negative breast cancer via extracellular vesicle reprogramming

MANUSCRIPT NUMBER: ONCSIS-20-0332R

CORRESPONDING AUTHORS NAME: Maria Felice Brizzi

PREVIOUS AUTHOR NAMES:

Tatiana Lopatina1, Cristina Grange1, Claudia Cavallari2, Victor Navarro-Tableros2, Giusy Lombardo1, Arturo Rosso1, Massimo Cedrino2, Margherita Alba Carlotta Pomatto1, Francesca Veneziano1, Isabella Castellano1, Giovanni Camussi1*, and Maria Felice Brizzi1*

UPDATED AUTHOR NAMES:

Malvina Koni has been included in the present version of the MS since she has performed biochemical and siRNA experiments requested by Reviewers

CHANGE TO AUTHOR LIST:

Tatiana Lopatina1, Cristina Grange1, Claudia Cavallari2, Victor Navarro-Tableros2, Giusy Lombardo1, Arturo Rosso1, Massimo Cedrino2, Margherita Alba Carlotta Pomatto1, Malvina Koni1, Francesca Veneziano1, Isabella Castellano1, Giovanni Camussi1*, and Maria

Print Name	Signature	Date
Giovanni Camussi	Giovanni Camussi	08/06/2020

SPRINGER NATURE

In accordance with Springer Nature Authorship Policy we agree to change the authors of the manuscript as indicated below.

NAME OF JOURNAL: Oncogenesis

TITLE OF MANUSCRIPT: Targeting IL-3Rα on tumor-derived endothelial cells blunts metastatic spread of triple negative breast cancer via extracellular vesicle reprogramming

MANUSCRIPT NUMBER: ONCSIS-20-0332R

CORRESPONDING AUTHORS NAME: Maria Felice Brizzi

PREVIOUS AUTHOR NAMES:

Tatiana Lopatina1, Cristina Grange1, Claudia Cavallari2, Victor Navarro-Tableros2, Giusy Lombardo1, Arturo Rosso1, Massimo Cedrino2, Margherita Alba Carlotta Pomatto1, Francesca Veneziano1, Isabella Castellano1, Giovanni Camussi1*, and Maria Felice Brizzi1*

UPDATED AUTHOR NAMES:

Malvina Koni has been included in the present version of the MS since she has performed biochemical and siRNA experiments requested by Reviewers

CHANGE TO AUTHOR LIST:

Tatiana Lopatina1, Cristina Grange1, Claudia Cavallari2, Victor Navarro-Tableros2, Giusy Lombardo1, Arturo Rosso1, Massimo Cedrino2, Margherita Alba Carlotta Pomatto1, Malvina Koni1, Francesca Veneziano1, Isabella Castellano1, Giovanni Camussi1*, and Maria

Print Name	Signature	Date
Maria Felice Brizzi	[signature]	08/06/2020
