# Supplementary material for: Targeting IL-3Rα on tumor-derived endothelial cells blunts metastatic spread of triple-negative breast cancer via extracellular vesicle reprogramming
Source: Oncogenesis. 2020 Oct 10;9(10):90. doi: 10.1038/s41389-020-00274-y (PMC7548009; doi:10.1038/s41389-020-00274-y)
Supplement: Supplementary file 3 — email authorship confirmation [file 41389_2020_274_MOESM3_ESM.pdf]

Da: **Tatiana Lopatina** <t.lopatina@gmail.com>
Date: lun 21 set 2020 alle ore 16:51
Subject: Re:
To: Maria Felice Brizzi <mariafelice.brizzi@unito.it>

I confirm my agreement to change the list of authors in the manuscript ONCSIS-20-0332R.

Sincerely,
Tatiana Lopatina

Da: **Cristina Grange** <cristina.grange@unito.it>
Date: lun 21 set 2020 alle ore 17:58
Subject:
To: Maria Felice Brizzi <mariafelice.brizzi@unito.it>

I confirm my agreement to change the list of authors in the manuscript ONCSIS-20-0332R.
Best regards
Cristina Grange

Da: **Claudia Cavallari** <cavallari.1184@gmail.com>
Date: lun 21 set 2020 alle ore 19:42
Subject: Re: firma
To: Maria Felice Brizzi <mariafelice.brizzi@unito.it>

I confirm my agreement to change the list of authors in the manuscript ONCSIS-20-0332R.

Claudia Cavallari

Da: **Victor Navarro** <victor.navarro@2i3t.it>
Date: mar 22 set 2020, 12:07
Subject: Signed document organog
To: Maria Felice Brizzi <mariafelice.brizzi@unito.it>

Dear Prof. Brizzi

I confirm my agreement to change the list of authors in the manuscript ONCSIS-20-0332R.

Kind regards,

Victor Navarro

Da: <lombardo88@hotmail.it>
Date: lun 21 set 2020 alle ore 17:18
Subject:
To: <mariafelice.brizzi@unito.it>

I confirm my agreement to change the list of authors in the manuscript ONCSIS-20-0332R

Giusy Lombardo

Da: **Arturo Rosso** <arturo.rosso@unito.it>
Date: lun 21 set 2020 alle ore 17:05
Subject: conferma
To: Maria Felice Brizzi <mariafelice.brizzi@unito.it>

I confirm my agreement to change the list of authors in the manuscript ONCSIS-20-0332R

--
Arturo Rosso

Da: **Massimo Cedrino** <massimo.cedrino@unito.it>
Date: lun 21 set 2020 alle ore 17:02
Subject: MC Oncogenesis
To: Maria Felice Brizzi <mariafelice.brizzi@unito.it>

I confirm my agreement to change the list of authors in the manuscript ONCSIS-20-0332R

Massimo Cedrino

Da: **Margherita Alba Carlotta Pomatto** <margheritaalbacarlotta.pomatto@unito.it>
Date: lun 21 set 2020 alle ore 17:16
Subject: Agreement authors ONCSIS-20-0332R.
To: Maria Felice Brizzi <mariafelice.brizzi@unito.it>

I confirm my agreement to change the list of authors in the manuscript ONCSIS-20-0332R.

Best regards,

Margherita Pomatto

Margherita Pomatto, Ph.D.
Department of Medical Science
Ospedale Molinette, Corso Dogliotti 14
10126 Torino
Tel. 011 633 6708

Malvina Koni <malvina.koni@unito.it> lun 21 set, 17:02 (19 ore fa)
a Maria

I confirm my agreement to change the list of authors in the manuscript ONCSIS-20-0332R

Da: <francescaveneziano11@gmail.com>
Date: lun 21 set 2020 alle ore 17:31
Subject: Author confitmation
To: <mariafelice.brizzi@unito.it>

I confirm my agreement to change the list of authors in the manuscript ONCSIS-20-0332R
Francesca Veneziano

Da: **Isabella Castellano** <isabella.castellano@unito.it>
Date: lun 21 set 2020 alle ore 17:25
Subject: paper oncogene
To: Maria Felice Brizzi <mariafelice.brizzi@unito.it>

Dear Prof Brizzi,
I confirm my agreement to change the list of authors in the manuscript ONCSIS-20-0332R.
Best Regards.

Isabella Castellano, M.D., PhD
Associate Professor of Pathology
Pathology Unit, Dept. Medical Sciences
University of Turin, Via Santena 7, 10126 Torino, Italy

Tel +390116634432
Fax +390116334633

Da: **Giovanni Camussi** <giovanni.camussi@unito.it>
Date: lun 21 set 2020 alle ore 18:08
Subject: Re:
To: Maria Felice Brizzi <mariafelice.brizzi@unito.it>

Dear Dr. Brizzi,
I confirm my agreement to change the list of authors in the manuscript ONCSIS-20-0332R.
Best regards
Giovanni Camussi

Da: **Maria Felice Brizzi** <mariafelice.brizzi@unito.it>
Date: lun 21 set 2020 alle ore 17:10
Subject: authrship
To: Maria Felice Brizzi <mariafelice.brizzi@unito.it>

I confirm my agreement to change the list of authors in the manuscript ONCSIS-20-0332R

Maria Felice Brizzi, MD PhD
Professore Associato di Medicina Interna
Dip. di Scienze Mediche
Università di Torino
c.so Dogliotti 14, 10126 Torino
tel. +39 011 6706653
mariafelice.brizzi@unito.it
